# In Silico Study of Superoxide Dismutase Gene Family in Potato and Effects of Elevated Temperature and Salicylic Acid on Gene Expression

**DOI:** 10.3390/antiox11030488

**Published:** 2022-02-28

**Authors:** Jelena Rudić, Milan B. Dragićević, Ivana Momčilović, Ana D. Simonović, Danijel Pantelić

**Affiliations:** Institute for Biological Research “Siniša Stanković”—National Institute of the Republic of Serbia, University of Belgrade, Bulevar despota Stefana 142, 11060 Belgrade, Serbia; jelena.rudic@ibiss.bg.ac.rs (J.R.); mdragicevic@ibiss.bg.ac.rs (M.B.D.); ivana.momcilovic@ibiss.bg.ac.rs (I.M.); ana.simonovic@ibiss.bg.ac.rs (A.D.S.)

**Keywords:** superoxide dismutase, potato, AlphaFold, heat stress, salicylic acid

## Abstract

Potato (*Solanum tuberosum* L.) is the most important vegetable crop globally and is very susceptible to high ambient temperatures. Since heat stress causes the accumulation of reactive oxygen species (ROS), investigations regarding major enzymatic components of the antioxidative system are of the essence. Superoxide dismutases (SODs) represent the first line of defense against ROS but detailed in silico analysis and characterization of the potato *SOD* gene family have not been performed thus far. We have analyzed eight functional *SOD* genes, three *StCuZnSOD*s, one *StMnSOD*, and four *StFeSOD*s, annotated in the updated version of potato genome (Spud DB DM v6.1). The *StSOD* genes and their respective proteins were analyzed in silico to determine the exon-intron organization, splice variants, cis-regulatory promoter elements, conserved domains, signals for subcellular targeting, 3D-structures, and phylogenetic relations. Quantitative PCR analysis revealed higher induction of *StCuZnSOD*s (the major potato *SOD*s) and *StFeSOD3* in thermotolerant cultivar Désirée than in thermosensitive Agria and Kennebec during long-term exposure to elevated temperature. *StMnSOD* was constitutively expressed, while expression of *StFeSOD*s was cultivar-dependent. The effects of salicylic acid (10^−5^ M) on *StSOD*s expression were minor. Our results provide the basis for further research on *StSOD*s and their regulation in potato, particularly in response to elevated temperatures.

## 1. Introduction

Potato (*Solanum tuberosum* L.) is the most important vegetable crop grown worldwide, essential for global food security. It is a cool-season vegetable, very susceptible to high ambient temperatures compared to other cultivated plants. Even mildly elevated temperatures (26–30 °C) may induce biochemical, physiological, and morpho-anatomical changes that affect the growth and development of this plant species [1,2,3]. High temperature can accelerate stem growth, reduce leaf area and reduce or inhibit root growth in potato [2]. The most prominent effects of high temperatures relate to the reduction in tuber induction, initiation and enlargement, and decrease in partitioning of dry matter to the tubers, which results in a decline in potato yield [2,3]. At the cellular level, high temperature disrupts membranes’ integrity, changes protein conformation, degrades the PSII component of the photosynthetic apparatus, and, due to impairment of electron transport chains in chloroplasts and mitochondria, promotes the production of reactive oxygen species—ROS [4,5]. Excessive production of ROS in plant cells can damage pigments, carbohydrates, lipids, proteins, nucleic acids, and in severe cases, induces cellular death [6]. On the other hand, ROS can act as signaling molecules that regulate many physiological processes during plant growth and development, and participate in various abiotic and biotic stress responses [7,8].

During evolution, an efficient defense antioxidant system developed in plants, encompassing enzymatic and non-enzymatic components that scavenge the toxic radicals and thus aid plants to cope with the large quantities of ROS. Superoxide dismutases (SOD, EC 1.15.1.1) are enzymes that catalyze the conversion or dismutation of toxic superoxide anion radical (O_2_^•−^) into hydrogen peroxide (H_2_O_2_) and oxygen (O_2_) and represent the first line of antioxidant defense against ROS [9]. They are metalloproteins, whose catalytic activity depends on the presence of metal prosthetic groups. In plants, SODs are classified into three groups based on their metal ions as cofactors: copper/zinc (CuZnSODs), manganese (MnSODs) and iron SODs (FeSODs). MnSODs and FeSODs share a high degree of amino acid sequence and structural homologies and are distinct from CuZnSODs [5]. Since phospholipid membranes are impermeable to charged O_2_^•−^ radicals, SOD isoforms, although encoded by nuclear genes, are distributed in different subcellular compartments [10]. CuZnSODs are mainly distributed in cytosol, peroxisomes, chloroplasts, and/or extracellular space, FeSODs are primarily located in the plastids, and MnSOD mainly occurs in the mitochondria and peroxisomes [5]. Plant CuZnSODs can be homodimeric (cytosolic) or homotetrameric (chloroplast and extracellular), built from ~15–17 kDa subunits. Similarly, FeSODs and MnSODs are either homodimeric or homotetrameric enzymes with the subunit size of 18–27 and 18–20 kDa, respectively [5]. Many findings indicate that SODs may play a significant role in the abiotic stress tolerance of plants, which is supported by the results of studies on transgenic plants overexpressing MnSOD and/or Cu/ZnSOD [11,12,13].

Salicylic acid (SA) is an essential endogenous growth regulator and signaling molecule in plants, which regulates different aspects of plant physiology. SA is involved in activating plant defense responses against biotic and abiotic stresses, including drought, chilling, heavy metal toxicity, heat and salinity [14]. The application of exogenous SA could improve thermotolerance in plants. The treatment with SA at a suitable concentration generally has an acclimation-like effect, leading to enhanced heat tolerance due to promotion of heat shock factor (HSF)–DNA binding [15], enhanced accumulation of heat shock proteins (HSP) [15,16] and modulation of the antioxidant enzyme activity in plants [17,18]. When applied during heat stress, SA may also alleviate adverse effects of elevated temperatures as observed in wheat where SA caused accumulation of proline and consequently improved net photosynthesis [19]. The application of exogenous SA increased SOD activity in *Digitalis trojana* [20], rhododendron [21], and tomato [18] during heat stress. The efficiency of SA as a protective or ameliorating agent against different stresses, however, depends on the plant species, developmental stage, the applied concentration, application method and endogenous SA level [22].

Despite the importance of potato as a staple crop, detailed in silico analysis and characterization of the *S. tuberosum* SOD gene family have not been performed so far. The previous version of the potato genome was assembled using short reads and represented only 86% of the 726 Mb—large genome of the doubled monoploid potato *S. tuberosum* L. Group Phureja DM 1–3 516 R44 [23]. An updated version of the same doubled monoploid clone genome (DM v6.1), with re-estimated size of 844 Mb, is available from 2020; it is based on the Oxford Nanopore Technologies long reads coupled with proximity-by-ligation scaffolding, yielding a chromosome-scale assembly [24]. Hereby we present an in depth in silico study of potato SOD (*StSOD*) genes retrieved from the updated genome version DM v6.1 that covers exon-intron organization, splice variants, cis-regulatory promoter elements, conserved domains, encoded proteins’ physicochemical properties, signals for subcellular targeting, prediction of 3D structures and phylogenetic relations with SODs from other plants. Due to global climate change, the rise in average ambient temperatures is predicted for most potato-growing regions in the 21st century [25], and investigations regarding the effects of prolonged mild heat stress on physiological, biochemical, and molecular responses of potato are gaining in importance [2]. Therefore, we analyzed *StSOD*s expression by reverse transcription quantitative PCR (qRT-PCR) after exposure of potato microplants to long-term mild heat stress (29 °C, three weeks) or slightly supraoptimal temperature treatment (26 °C, three weeks), with and without exogenous SA. Our results provide the basis for further research of *StSOD*s and can be important for a better understanding of potato antioxidant system response to elevated temperature and SA.

## 2. Materials and Methods

### 2.1. Potato Genomic Resources

Potato (*Solanum tuberosum* L.) SOD gene data, including accession numbers, chromosomal location, sequences, corresponding transcripts and proteins sequences were based on the doubled monoploid *S. tuberosum* Group Phureja DM 1-3 516 R44 and were retrieved from Spud DB Potato Genomics Resources (DM v6.1, http://spuddb.uga.edu (accessed on 15 April 2021).

### 2.2. StSOD Promoter Analysis

Analysis of transcription factor (TF) binding sites was performed on promoters of *StSOD*s encompassing a region 1000 nt upstream and 100 nt downstream from the transcription start site. Binding site prediction was performed using Plant Transcriptional Regulatory Map (PlantRegMap, http://plantregmap.gao-lab.org/binding_site_prediction.php, accessed on 20 June 2021) [26] with a *p*-value threshold of 10^−5^. All binding sites with a q-value < 0.05 [27] were retained. TFs that bind identified motifs were found based on the *S. tuberosum* TF list (http://planttfdb.gao-lab.org/download.php, accessed on 20 June 2021). Gene ontology (GO) annotation of TFs was attained from Plant Transcription Factor Database v5.0 (PlantTFDB v5.0, http://planttfdb.gao-lab.org/index.php, accessed on 20 June 2021) [28]. Alternative analysis of *StSOD* promoters with PlantCARE [29] is presented only in supplementary materials.

### 2.3. In Silico Characterization of StSOD Protein Features

StSOD proteins features, including molecular weight, theoretical isoelectric point (pI), instability index and aliphatic index were computed using ProtParam tool (https://web.expasy.org/protparam, accessed on 22 June 2021) [30]. Subcellular localization of StSOD proteins was predicted using a combination of tools, including DeepLoc-1.0 (https://services.healthtech.dtu.dk/service.php?DeepLoc-1.0, accessed on 1 July 2021) [31], CELLO v2.5 (http://cello.life.nctu.edu.tw, accessed on 1 July 2021) [32], Light Attention (LA, https://embed.protein.properties/, accessed on 1 October 2021) [33], TargetP2 (https://services.healthtech.dtu.dk/service.php?TargetP-2.0, accessed on 1 July 2021) [34], PTS1 Predictor (https://mendel.imp.ac.at/pts1/, accessed on 2 July 2021) [35,36] and PredPlantPTS1 (http://ppp.gobics.de, accessed on 2 July 2021) [37]. Protein family (Pfam, http://pfam.xfam.org, accessed on 27 June 2021) [38] annotation was performed using hmmer 3.3.2 [39] and Pfam34 database (http://ftp.ebi.ac.uk/pub/databases/Pfam/releases/Pfam34.0/, accessed on 27 June 2021). Conserved Domain Database (CDD, https://ncbi.nlm.nih.gov/Structure/cdd/cdd.shtml, accessed on 7 September 2021) [40] annotation was performed using CD search online web server (https://ncbi.nlm.nih.gov/Structure/cdd/wrpsb.cgi, accessed on 7 September 2021) [41]. StSOD sequences processed based on TargetP2 transit peptide (or target peptide, TP) predictions were used for multiple sequence alignments and tertiary protein structure prediction. Multiple sequence alignments were generated with the DECIPHER R package [42] using 10 iterations and 10 refinements and other default options. Percent identity between aligned protein sequences was calculated as (identical positions)/(aligned positions + internal gap positions) × 100%. Tertiary protein structure was estimated using AlphaFold [43] queried via UCSF ChimeraX 1.3 [44]. The generated best structure per *StSOD* sequence was assessed using MolProbity 4.4 [45] via SWISS-MODEL Workspace (https://swissmodel.expasy.org/assess, accessed on 28 October 2021) [46] and rendered using UCSF ChimeraX 1.3. Ramachandran diagrams were created using the R package ggrama [47].

### 2.4. Phylogenetic Analysis of StSOD Protein Sequences

Phylogeny of StSOD proteins was assessed by comparison with homologs from 18 plant species: *Arabidopsis thaliana*, *Beta vulgaris*, *Capsicum annuum*, *Daucus carota*, *Glycine max*, *Helianthus annuus*, *Nicotiana attenuata*, *Phaseolus vulgaris*, *Solanum lycopersicum*, *Manihot esculenta*, *Oryza sativa*, *Saccharum spontaneum*, *Zea mays*, *Sorghum bicolor*, *Hordeum vulgare*, *Ananas comosus*, *Musa acuminata* and *Dioscorea rotundata*. Proteomes from these species were obtained from Ensembl release 49 (https://plants.ensembl.org/, accessed on 1 March 2021, http://ftp.ensemblgenomes.org/pub/plants/release-49/fasta/, accessed on 1 March 2021). Raw bit-score top hits per *StSOD* sequence per plant were filtered and the top seven homologs per *StSOD* sequence were used for phylogenetic analysis. Sequence alignment was performed with the DECIPHER R package [42] using 10 iterations and 10 refinements and other default options. Uninformative sites in the alignment were removed with DECIPHER R package with default options using the small-sample size correction [48]. The resulting alignment was used for fitting a maximum likelihood tree using the LG model of amino acid replacement [49]. The fitting was performed with optimization of the gamma rate parameter and proportion of invariable sites using stochastic rearrangement starting from the neighbor-joining tree using phangorn R package [50]. To assess cluster stability, non-parametric bootstrap was performed for 100 iterations. For rooting of the CuZnSOD phylogenetic tree, the CuZnSOD protein sequence from *Saccharomyces cerevisiae* was used (PDB: 1JK9). No suitable sequence was found for rooting of the Mn-FeSOD phylogenetic tree, so it was midpoint rooted.

### 2.5. Plant Material and Growth Conditions

Commercial *S. tuberosum* L. cultivars, Agria, Désirée and Kennebec, were used in experiments. The three unrelated potato varieties were selected to validate the presence of investigated genes and compare *StSOD*s expression in tetraploid genotypes that differ in heat tolerance. Based on our unpublished data, Désirée was considered relatively heat tolerant, Kennebec as moderately sensitive, and Agria as heat-sensitive genotype.

Virus-free tubers of three potato cultivars were obtained from Solanum Komerc, Guča, Serbia. In vitro cultures were established from surface-sterilized sprouts, which were transferred on the basal medium (BM) consisting of Murashige and Skoog macro and micro-mineral salts [51], Linsmaier and Skoog vitamins [52], 0.7% agar, 3% sucrose, 100 mgL^−1^ myo-inositol and supplemented with 0.5 mgL^−1^ 6-benzylaminopurine (BAP; Sigma Aldrich, St. Louis, MO). Shoots obtained on this medium gave rise to plantlets when transferred to BM without BAP. Microplants were grown in a controlled environment (21 °C, 16 h light period, light flux 90 μmol m^−2^ s^−1^) and were routinely subcultured every four weeks on BM using single-node stem cuttings (SNC).

### 2.6. SA and Temperature Treatments

SNCs (10–15 mm) from four-week-old potato microplants were transferred on BM or BM supplemented with 10^−5^ M SA in glass jars (10 SNCs per jar) with vented polypropylene caps. SA (Sigma Aldrich, St. Louis, MO, USA) was dissolved in 96% ethanol and added to the medium before the sterilization at 114 °C for 25 min, while the equivalent volume of ethanol was added in control. Based on our preliminary testing of SA concentrations in the range 10^−4^–10^−6^ M and literature data [53], the particular SA concentration was selected as the best for alleviating adverse effects of heat treatments on microplants’ growth and development. The explants were grown in the growth chamber (Aralab, Rio de Mouro, Portugal) under 16 h photoperiod, light flux 90 μmol m^−2^ s^−1^, 70% humidity, and at three different temperatures: 21 °C, 26 °C or 29 °C. After three weeks, fully developed leaves were collected from plants grown in four jars (one biological sample), frozen in liquid nitrogen before storage at −80 °C and further used for RNA extraction. Three biological samples were used for qRT-PCR analysis.

### 2.7. RNA Extraction and cDNA Synthesis

Total RNA was extracted from 0.5 g of frozen potato leaves using TRIzol reagent (Invitrogen, Carlsbad, CA, USA) following the manufacturer’s instructions, and stored at −80 °C until use. RNA quality and concentration were determined using NanoPhotometer N60 (Implen, Munich, BY, Germany), and all RNA samples were diluted to 1 µg µL^−1^ in RNase-free water. The purity of RNA was gauged by the absorbance ratio of A260/A280. The integrity of isolated RNA was checked electrophoretically and assessed by the ratio of 28S/18S rRNA. The genomic DNA contamination was eliminated from the total RNA by DNase I treatment (Fermentas, Hanover, MD, USA), and the first-strand cDNA was synthesized from 3 µg of RNA using Revert Aid First Strand cDNA Synthesis Kit (Fermentas, Hanover, MD, USA) with oligo(dT) primers according to manufacturer’s instructions.

### 2.8. qRT-PCR Analysis

Oligonucleotide primers for the amplification of *StSOD* transcripts were designed using Primer-BLAST available at NCBI (https://ncbi.nlm.nih.gov/tools/primer-blast/, accessed on 10 July 2020), while the primer pair for *StCuZnSOD2* was synthesized according to [54]. For *StSOD* genes with multiple gene models (splice variants, see Table 1), the primers were designed to amplify all models of one gene (Table S1). Primers specificity was checked by PCR followed by electrophoretic sizing and by melting curve analysis. The obtained amplicons were purified from the agarose gels using Gene JET™ Gel Extraction Kit (Thermo Fisher Scientific, Waltham, MA, USA), quantified using NanoPhotometer N60 (Implen, Munich, BY, Germany), and serially diluted in a 10^9^–10^2^ copies µL^−1^ range to be used as standards for absolute qPCR quantification. The qPCR was run in three technical replicates for each biological replicate on MicroAmp™ Optical 96-Well Plates (Thermo Fisher Scientific, Waltham, MA, USA), in 10 µL reaction mixtures comprising of cDNA corresponding to 100 ng of total RNA, forward and reverse primers (7.5 µM each), and 5 µL Maxima SYBR Green/ROX qPCR Master Mix (2x) (Thermo Fisher Scientific, Waltham, MA, USA). The amplification was conducted using Applied Biosystems QuantStudio™ 3 Real-Time PCR system (Thermo Fisher Scientific, Waltham, MA, USA), with the program: initial denaturation at 95 °C for 10 min, followed by 40 cycles at 95 °C for 15 s and 60 °C for 1 min. Absolute expression values were normalized against the averaged expression data of the internal control genes *60SL36* and *CYC* [55].

### 2.9. Statistical Analysis

Statistical analysis was performed using IBM SPSS Statistics version 25 (International Business Machines Corporation, Armonk, NY, USA). Levene’s test was used to verify the homogeneity of variances of the data set. One-way analysis of variance was performed with multiple comparisons analysis of means by either Tukey’s HSD (for equal variances) or Dunnett’s T3 (for unequal variances) post-hoc test at a significance level of 0.05. The data were shown as mean values ± standard deviation (S.D.).

## 3. Results

### 3.1. StSOD Genes: Structure and Chromosomal Distribution

Ten potato genes, annotated as *SOD*, were derived from the Spud DB Potato Genomics Resources—DM v6.1. Two genes, Soltu.DM.10G011570 and Soltu.DM.06G013120, were not further investigated, since they do not encode full-length functional SOD proteins, but ~100 amino acids long polypeptides which have similarity with SOD sequences. A copper chaperone gene, Soltu.DM.08G026370, also encodes a protein that contains a copper/zinc superoxide dismutase domain (Pfam: PF00080), but it lacks crucial active site residues to be a CuZnSOD, so it was also omitted. In total, eight full-length *StSOD* genes: three *StCuZnSOD*s, one *StMnSOD*, and four *StFeSOD*s, were the object of our study (Table 1). The eight analyzed *StSOD*s are physically located on five out of 12 chromosomes: *StCuZnSOD1*, *StCuZnSOD2*, and *StFeSOD3* are on chromosomes 1, 11, and 2, respectively; two genes, *StCuZnSOD3* and *StFeSOD2*, are located on chromosome 3, while three genes, *StMnSOD*, *StFeSOD1*, and *StFeSOD4*, are on chromosome 6. *StFeSOD1* and *StFeSOD4*, separated by only 1566 bp, are considered tandemly duplicated [56].

**Figure 1:**
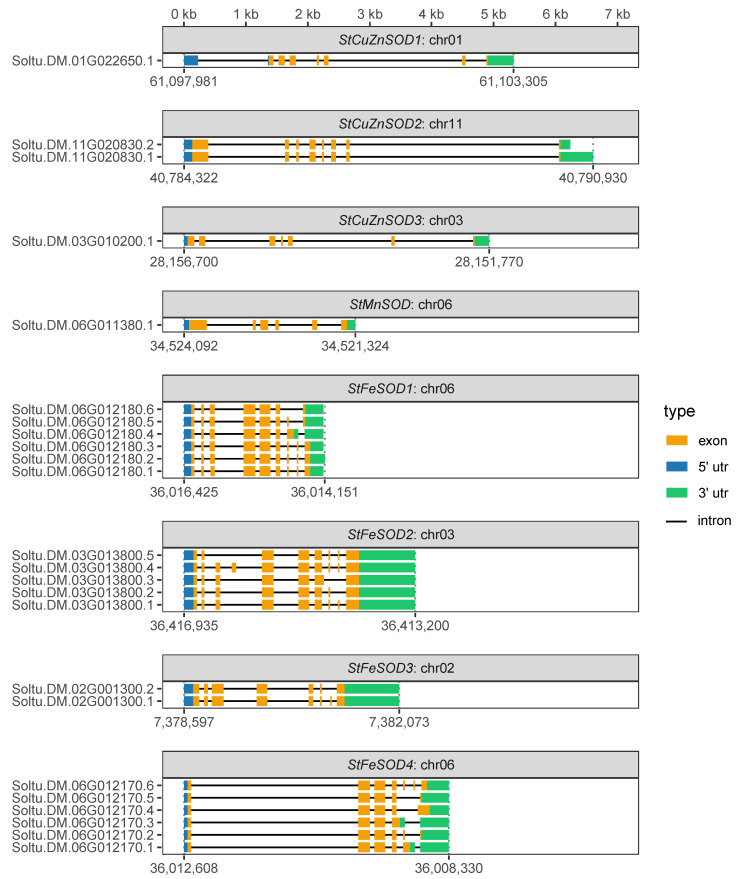
Exon-intron organization of *StSOD* genes. The blue and green rectangles represent UTR, the orange rectangle represent exons, and black lines represent introns.

As listed in Table 1, *StCuZnSOD2* (Soltu.DM.11G020830) and all *StFeSOD*s (Soltu.DM.06G012180, Soltu.DM.03G013800, Soltu.DM.02G001300, Soltu.DM.06G012170) have at least two and up to six transcripts. Alignment of these transcripts against their genes revealed that different gene models vary in the number of exons and introns or, in some cases, in the length of the 3′-UTR (Figure 1), suggesting that these are alternative splice variants. The differences in exon-intron organization and UTRs among *StSOD* genes and their splice variants are depicted in Figure 1, where can be seen that *StSOD* gene models possessed between 4 to 9 introns and 5 to 10 exons. *StFeSOD1* and *StFeSOD2* genes share similar exon/intron organization patterns, but with differences in exons/introns lengths, while the rest of *StSOD* genes exhibited disparate exon/intron structures.

### 3.2. Analysis of Promoter Regions of StSOD Genes

**Figure 2:**
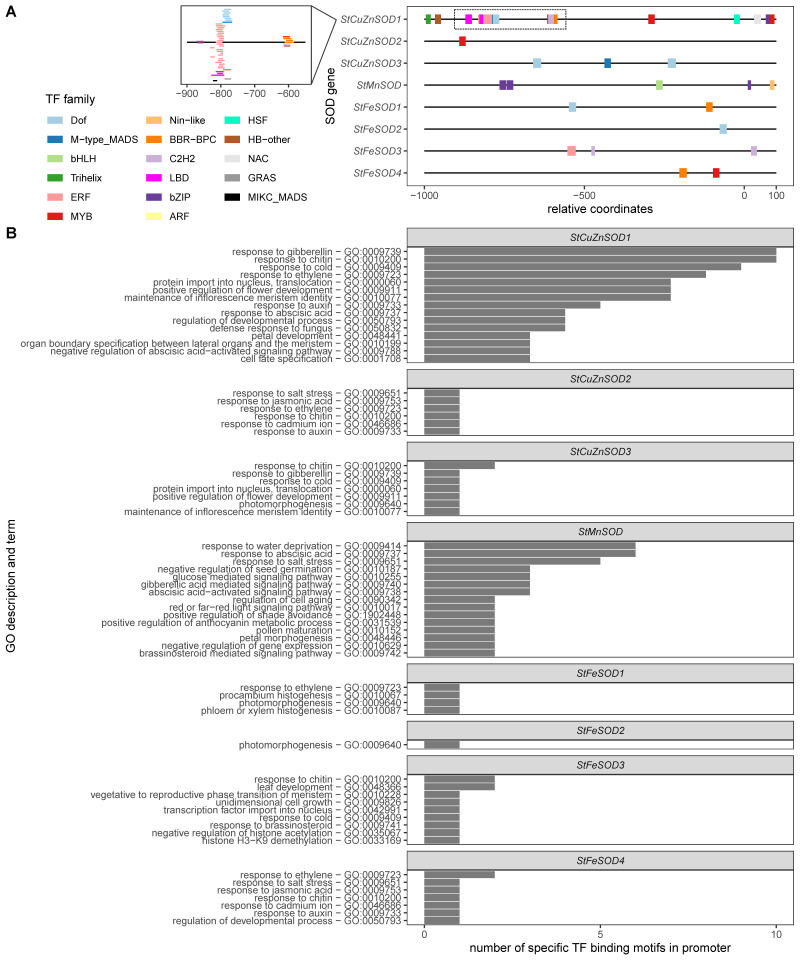
Transcription factor binding motifs in promoters of *StSOD* genes. (**A**) Location of binding sites for various TF families (color legend). (**B**) Biological process gene ontology terms associated with specific potato TFs which bind the identified motifs. Only unique GO terms per binding motif were counted. Non-informative terms (such as “process regulation of transcription, DNA-templated” and similar) present in all or most TF families were omitted.

The potential regulation of *StSOD* genes was investigated by identification of TF binding motifs (cis-regulatory elements) in the *StSOD*s promoter regions. Based on the binding analysis using PlantRegMap [26], members of 17 TF families may bind to the eight StSOD (Figure 2A and Table S2).

The gene with the highest number of cis-regulatory elements is *StCuZnSOD1*, which contains binding motifs for 15 different TF families (Figure 2A). An especially motif dense region is located ~800 nt upstream of the transcription start site of *StCuZnSOD1*, which contains binding motifs for Dof, M-type_MADS, ERF, BBR-BPC, LBD, ARF, GRAS and MIKC_MADS TF families (Figure 2A, insert). In contrast, the *StCuZnSOD2* promoter has a single MYB binding region, while *StCuZnSOD3* contains two Dof and one M−type_MADS binding region (Figure 2A). *StMnSOD* contains three bZIP binding regions, a single bHLH and Nin-like binding sequence motif, while the remaining *StFeSOD* genes contain one to three binding regions for different TF families (Figure 2A).

To gain insight into the potential biological roles of the identified cis-regulatory elements, the biological process GO terms associated with specific potato TFs which bind the identified motifs were summarized per *StSOD* promoter and binding motif (Figure 2B and Table S3). The GO terms were obtained from PlantTFDB and non-informative terms (such as “process regulation of transcription, DNA-templated” and similar) were omitted. Based on TF-associated GO terms, *StCuZnSOD1* is regulated in response to external stressors such as cold and fungal infection, by several plant hormones, including gibberellin, ethylene, auxin and abscisic acid, and during flower development. *StCuZnSOD3* is to some extent similar in this regard to *StCuZnSOD1* (regulation by cold, gibberellins, chitin, flower development) because its promoter contains binding sites for M−type_MADS and Dof TFs, mediators of these types of regulation. *StCuZnSOD2* contains a single MYB binding cis-element associated with GO terms: response to salt stress, ethylene, auxin, jasmonic acid, chitin and cadmium concentration. The *StMnSOD* gene is regulated in response to desiccation, salt stress, abscisic and gibberellic acids, brassinosteroid and phytochrome signaling pathways. *StFeSOD1* contains binding motifs for Dof and BBR-BPC TFs and is regulated during vascular system development (Dof) and in response to ethylene (BBR-BPC). *StFeSOD2* contains a single Dof TF binding element responsive during light-mediated development. *StFeSOD3* contains an ERF and two C2H2 TFs; ERF is associated with response to chitin, cold and leaf development GO terms, while C2H2 is associated with meristem transition, unidimensional cell growth and response to brassinosteroid. *StFeSOD4* contains a BBR-BPC binding element associated with response to ethylene and regulation of developmental processes, and a MYB binding element associated with response to salt stress, chitin, ethylene, auxin and jasmonic acid.

### 3.3. Characteristics of StSOD Proteins

Physicochemical properties of StSOD proteins, including their length, isoelectric point (pI), instability index, aliphatic index, and molecular weights (MW), are shown in Table 1. Different StSODs are 152–304 amino acids long, with MW ranging from 15.3 to 37.6 kDa.

Theoretical pI values of StSODs are in a 5.20–7.85 range, indicating that almost all members of StSODs are acidic, except for StMnSOD and two StFeSODs, which are slightly basic. Based on the aliphatic index, which may be regarded as a positive factor for the enhancement of thermostability of globular proteins [57], the most thermostable protein is StMnSOD, which has the highest aliphatic index (91.14), while StCuZnSODs, with an average aliphatic index of 85.59 are generally more thermostable than StFeSODs (average aliphatic index of 76.35). The values of the instability index determine the stability of the protein in a test tube. A value of instability index above 40 predicts that the protein may be unstable. Almost all SODs were predicted to be stable (<40), except StFeSOD2 and StFeSOD3.

### 3.4. StSOD Protein Structure and Subcellular Localization

**Figure 3:**
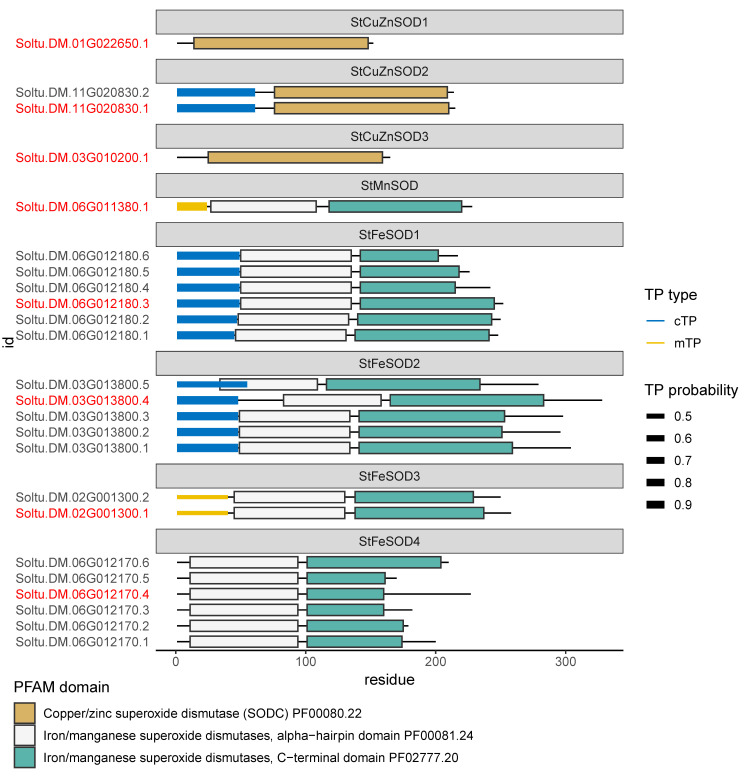
Annotation of protein sequences encoded by *StStSOD* genes. Protein sequences from all gene models are shown; protein products from representative (according to Spud DB DM v6.1 annotation) gene models are highlighted in red. Pfam domains are indicated by the color legend. TargetP2 annotation of target peptides (TP) is indicated by a blue (mitochondria target peptide—mTP) or yellow (chloroplast target peptide—cTP) segment on the N-terminal side. The width of the segment representing the TP corresponds to TargetP2 TP probability.

To gain insights into the organization of StSOD proteins structure we performed domain annotation using Pfam database, along with annotation of predicted N-terminal target peptides (Figure 3) and subcellular localization based of four algorithms: TargetP2, DeepLoc, CELLO and Light Attention (LA, Table 2). This was done for protein sequences translated in silico from all annotated StSOD gene models. The CuZnSOD protein sequences contain one Pfam domain characteristic for this class of proteins (PF00080.22), while MnSOD and FeSODs contain two Pfam domains: the N-terminal Iron/manganese SOD α-hairpin domain (PF00081.24) and the Iron/manganese SOD C-terminal domain (PF02777.20, Figure 3).

Proteins StCuZnSOD2, StFeSOD1 and StFeSOD2 are predicted to be targeted to chloroplast by all four tools used (Table 2, Figure 3). StMnSOD is predicted to be mitochondria-targeted. Analysis by TargetP2 indicates that StCuZnSOD1 and StCuZnSOD3, as well as StFeSOD4 proteins are without predicted mitochondrial (mTP), chloroplastic (cTP) or secretory N-terminal transit peptides, so it is likely that these proteins are located in the cytosol or a cellular compartment other than mitochondria or chloroplasts (Table 2, Figure 3). The recently released subcellular localization prediction algorithm LA, based on embeddings from protein sequence language models, places the protein products of *StCuZnSOD1* and *StFeSOD4* genes in the cytosol, while the *StCuZnSOD3* product is predicted to be located in the peroxisomes. However, peroxisome-specific tools PTS1 Predictor and PredPlantPTS1 do not localize StCuZnSOD3 in the peroxisomes (Table 2). The most probable subcellular localization of isoforms StCuZnSOD3, StFeSOD3 and StFeSOD4, for which different tools gave different predictions, is discussed later in the context of literature data and phylogenetic relations.

### 3.5. Predicted Tertiary Structure of Potato SODs

The structural features of StSOD proteins corresponding to representative gene models (as annotated within Spud DB DM v6.1) were evaluated after tertiary structure prediction using state-of-the-art method AlphaFold. The obtained structures were compared with experimentally determined SODs from the Protein Data Bank (PDB, https://www.rcsb.org/, accessed on 16 November 2021). Proteins with predicted TP were processed prior to submission to AlphaFold. The AlphaFold models of StSOD proteins are characterized by the high percentage of Ramachandran favored residues (median: 97.8%), a low percentage of Ramachandran outliers (median: 0%), and low clash score (the number of serious clashes per 1000 atoms; median: 0.94, Table 3 and Supplementary Info S7). The structure with the highest percentage of Ramachandran outliers was StCuZnSOD3 (Soltu.DM.03G010200.1); however, the majority (3/4) of these outliers were in the first 12 *N*-terminal amino acids, a region predicted not to be in secondary structures by AlphaFold (Table 3).

**Figure 4:**
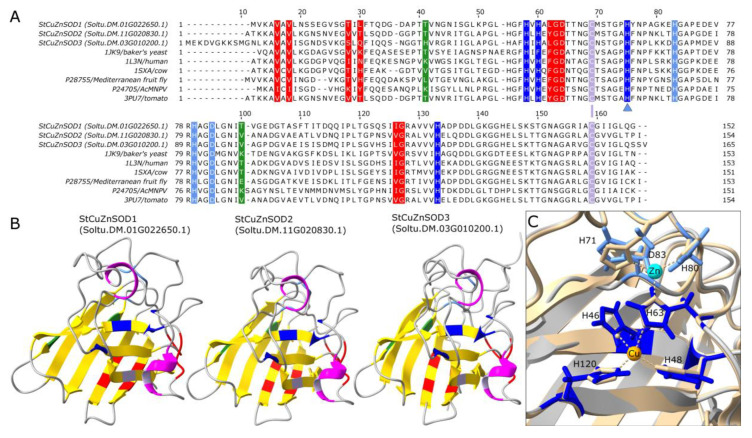
Structural features of StCuZnSODs. (**A**) Multiple sequence alignment of StCuZnSODs with several sequences of the same class with experimentally determined PDB structures. Processed StCuZnSOD sequences are shown based on TargetP2 transit peptide prediction. Residues are annotated according to CDD accession cd00305 (Cu/Zn_Superoxide_Dismutase): E-class dimer interface (eukaryotic polypeptide binding site) residues are colored red; P-class dimer interface (prokaryotic polypeptide binding site) residues are colored green; residues involved in Cu ion binding are colored blue, and residues involved in Zn ion binding are colored light blue. The arrow indicates the residue which is involved in binding of both Cu and Zn ions. Two cysteine residues involved in a disulfide bond as well as a bond itself (line) are colored violet. (**B**) AlphaFold models of StCuZnSODs: β-strands are colored yellow, α-helices are colored magenta, specific residues are colored as in A. (**C**) Metal binding site of StCuZnSOD2: the AlphaFold model of StCuZnSOD2 (in grey) was 3D aligned with 3PU7 (in tan) PDB structure (experimentally determined PDB structure of the tomato CuZnSOD). Metal ion position and coordinate bonds are based on 3PU7 Biological Assembly 1. StCuZnSOD2 residues involved in Cu and Zn ion binding are colored blue and light blue, respectively, and labeled according to processed peptide position. H63 residue is involved in binding of both metal ions.

CuZnSOD protein sequences share high similarities across species belonging to different kingdoms of life. The high sequence identity is mirrored in the extraordinary similarity of tertiary structures of predicted AlphaFold models of StCuZnSODs, both among each other, as well as to experimentally determined CuZnSOD proteins from the PDB (Figure 4B,C). The eight-stranded Greek key β-barrel fold, characteristic of the eukaryotic (E-class) CuZnSODs [58], clearly describes the obtained StCuZnSOD structures (Figure 4B). The conserved residues involved in E-dimer interface (colored red in Figure 4) are located in β1 and β2 (labeled from the N-terminus) and in the coils connecting β4 to β5 and β6 to β7. The aforementioned Greek key motif is formed by β3–β6. The metal ion binding sites of CuZnSODs contains six conserved H and one conserved D. The Cu binding site consists of four H residues, while one of these H also contributes to Zn binding (Figure 4A,C). Two H residues involved in Cu ion binding are located in β4 (H46 and H48 in StCuZnSOD2, when amino acid numbering excludes the 61 amino acid-long cTP). All the residues involved in Zn ion binding (including the H involved in both Cu and Zn ion binding) are located in the loop connecting β4 and β5 (H63, H71, H80, and D83 in StCuZnSOD2), while the remaining H residue involved in Cu binding (H120 in StCuZnSOD2) is located in β7 (Figure 4B,C). The superimposition of 3D aligned structures of the StCuZnSOD2 model and the experimentally determined structure of the closely related tomato CuZnSOD (PDB: 3PU7) clearly demonstrates the conserved orientation of the active site residues (Figure 4C).

**Figure 5:**
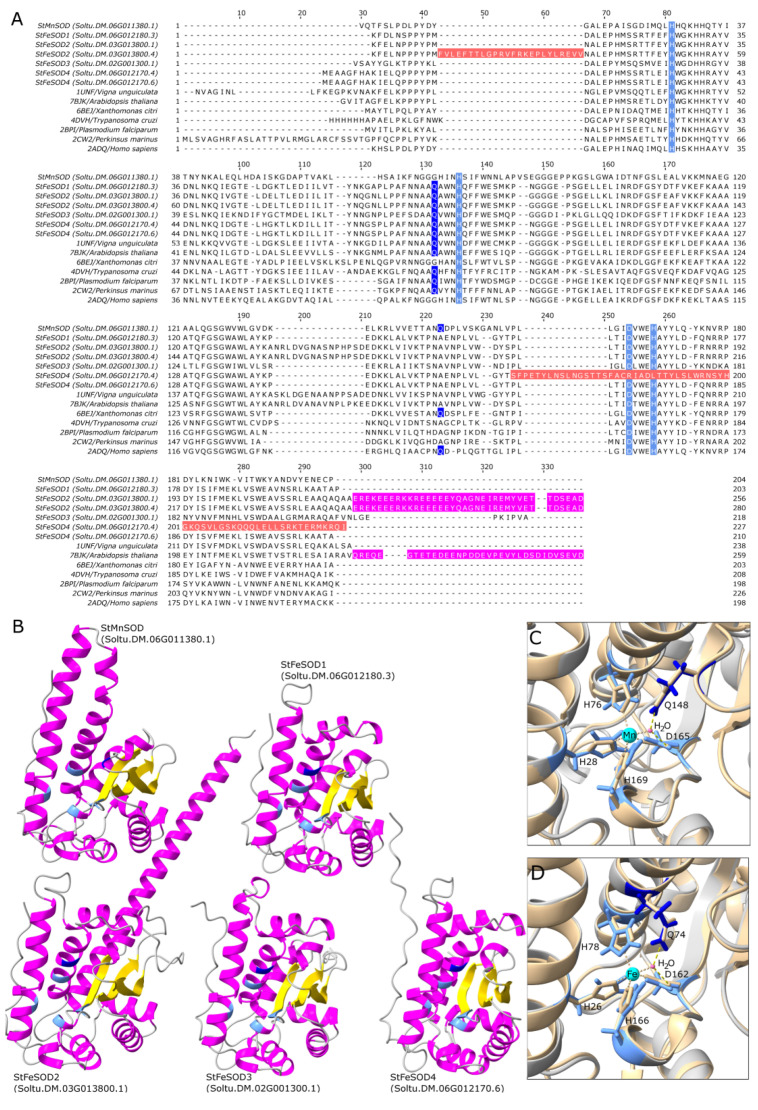
Structural features of StMnSOD and StFeSODs. (**A**) Multiple sequence alignment of potato Mn-FeSODs with several sequences of the same class with experimentally determined PDB structures. Processed potato Mn-FeSOD sequences are shown based on TargetP2 transit peptide prediction. Residues involved in metal ion binding are colored light blue; residues involved in hydrogen-binding of the water molecule, which acts as the fifth metal ion ligand, are colored blue; Annotation is based on PDB structures 2ADQ, 1UNF and 7BJK. The abnormal peptide regions of two representative StFeSOD sequences, Soltu.DM.06G012170.4 (StFeSOD4) and Soltu.DM.03G013800.4 (StFeSOD2) are colored red. The long C-terminal tail of Soltu.DM.03G013800.1 and Soltu.DM.03G013800.4 is colored magenta. (**B**) AlphaFold models of StMnSOD and StFeSODs: β-strands are colored yellow, α-helices are colored magenta, specific residues involved in metal ion binding are colored as in A. (**C**) Metal binding site of StMnSOD: the AlphaFold model of StMnSOD (in grey) was 3D aligned with 2ADQ (in tan) PDB structure (experimentally determined PDB structure of the human MnSOD). Mn ion position, coordinate bonds (tan) and hydrogen-bonds (yellow) stabilizing the water molecule (magenta) which is the fifth coordination partner of the metal ion are based on 2ADQ Biological Assembly 1. StMnSOD residues involved in metal ion binding are colored light blue and labeled according to processed peptide position. The water molecule is stabilized with hydrogen-bonds (yellow) from StMnSOD Q148 (blue) and D165 (light blue). (**D**) Metal binding site of StFeSOD1: the AlphaFold model of StFeSOD1 (in grey) was 3D aligned with 7BJK (in tan) PDB structure (experimentally determined PDB structure of the chloroplastic FeSOD PAP9 from *Arabidopsis thaliana*). Metal ion position (Fe), coordinate bonds (tan) and hydrogen-bonds (yellow) stabilizing the water molecule (magenta) which is the fifth coordination partner of the metal ion are based on 7BJK Biological Assembly 1. StFeSOD1residues involved in metal ion binding are colored light blue and labeled according to processed peptide position. The water molecule is stabilized with hydrogen-bonds from StFeSOD1 Q74 (blue) and D162 (light blue).

The initial alignment of representative potato Mn-FeSOD protein sequences (Figure 5A) indicated that two sequences corresponding to the representative gene models Soltu.DM.03G013800.4 (*StFeSOD2* gene) and Soltu.DM.06G012170.4 (*StFeSOD4* gene) contain abnormal regions (emphasized by the red color in MSA—Figure 5A). Soltu.DM.03G013800.4 contains a 24 amino acid-long insert at the N-terminal side, which is not present in other Mn-FeSODs, while Soltu.DM.06G012170.4 contains an aberrant C-terminal region of over 60 amino acids lacking two conserved residues involved in metal ion coordination (Figure 5A). This is the reason the Iron/manganese SOD C-terminal domain is shorter in this protein sequence (Figure 3) as compared to other Mn-FeSODs. Therefore, it appears that the criterion used for establishing which sequences are representative in Spud DB DM v6.1 is sequence length; in other words, the gene model coding the longest protein sequence is used as the representative gene model by default, without taking into account sequence structural features. Based on our observations the representative sequence for StFeSOD4 gene should be Soltu.DM.06G012170.6 instead of Soltu.DM.06G012170.4, as the longest protein sequence containing full-length PF00081 and PF02777 Pfam domains (Figure 3), and all characteristic amino acids involved in metal ion binding (Figure 5A). The representative sequence for *StFeSOD2* gene should be Soltu.DM.03G013800.1 instead of Soltu.DM.03G013800.4, because it contains the full-length PF00081 and PF02777.20 Pfam domains but lacks the non-characteristic 24 amino acid long N-terminal insert (Figure 5A). Hence, we used these sequences for tertiary structure modeling, along with StMnSOD and the default representative sequences for StFeSOD1 and StFeSOD3 (Table 1 and Table 3 and Figure 5B).

The obtained AlphaFold models of the five potato Mn-FeSODs display a similar fold to experimentally determined Mn-FeSODs from other organisms. The ~100 amino acid long α-hairpin domain of all StFeSODs consists of three α-helices, while the α-hairpin domain of the StMnSOD consists of two long α-helixes (Figure 5B). The C-terminal domain of all five potato Mn-FeSODs incorporates a central antiparallel β-sheet with three β-strands, which is surrounded by α-helices. In addition to the structural features common to all StFeSODs, StFeSOD2 is characterized by a long negatively charged C-terminal region rich in E/D (annotated with magenta color in Figure 5A) just after the Iron/manganese SOD C-terminal domain.

The metal binding site of Mn-FeSOD protein sequences consists of three conserved H and one conserved D residue (H28, H76, D165 and H169 in StMnSOD, where the amino acid numbering excludes the 24 amino acid-long mTP, Figure 5A,C). H28 and H76 (StMnSOD) are located in the first and last helix of the α-hairpin domain respectively while D165 and H169 (StMnSOD) are located in the C-terminal domain, specifically at the end of the third beta strand (β3) and in the short helical region just after β3 respectively. The metal ion in the catalytic center of Mn-FeSODs is penta-coordinated by these four conserved residues and a water molecule [59,60]. This ligand water molecule is stabilized by hydrogen-bonds with the D residue, also involved in metal ion coordination, and a conserved Q residue (annotated with dark blue color in Figure 5A,B). In FeSOD sequences the water-stabilizing Q is within the last helix of the α-hairpin domain (Q74 in StFeSOD1), while in MnSODs the Q (Q148 is StMnSOD) is located in the coil connecting β2 and β3 of the C-terminal domain (Figure 5A,C). Comparison of the active sites of StMnSOD with human MnSOD (PDB: 2ADQ, Figure 5C) as well as StFeSOD1 with Arabidopsis FeSOD (PAP9, Figure 5D) indicates conservation of active site residue orientation.

### 3.6. Phylogenetic Relations of StSOD Proteins

**Figure 6:**
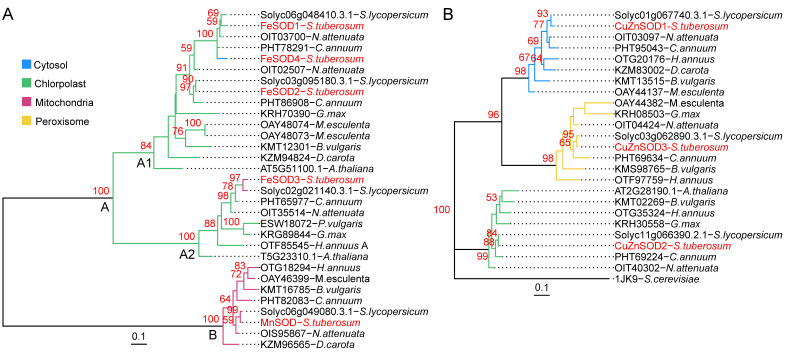
Maximum likelihood phylogenetic trees of StSODs. (**A**) Midpoint rooted Mn-FeSOD phylogenetic tree. (**B**) Rooted CuZnSOD phylogenetic tree. Support values were obtained using 100 iterations of non-parametric bootstrap; values over 50/100 are indicated with a red number. Clade/tip coloring is based on subcellular location (color legend) obtained using Light Attention server.

Phylogenetic relations of StSOD protein sequences were estimated by comparison with homologues from cultivated plant species and model organisms such as Arabidopsis. The phylogenetic trees were estimated independently for the Mn-FeSODs and CuZnSODs, because even though these two classes share some common features, these are far too sparse to produce a reliable phylogenetic model (Figure 6). The midpoint rooted maximum likelihood phylogenetic tree of potato Mn-FeSODs (Figure 6A) clearly indicates the presence of two clades, A and B, which are quite phylogenetically distant, so no suitable outgroup sequence for rooting of the tree could be found (all attempted outgroups clustered either with sequences from A or sequences from B therefore a midpoint rooted tree is presented). Cluster B is formed from MnSOD sequences homologous to StMnSOD. All of these sequences contain predicted mTP. In addition, these sequences contain the characteristic Q residue, involved in hydrogen binding with the water molecule ligand, located in the C-terminal domain (the informative sites of the Mn-FeSODs alignment used for the construction of the phylogenetic tree are provided in Figure S2, where the mentioned Q residue is colored blue). In all sequences in clade A, this Q is located in the α-hairpin domain (Figure S2). Clade A contains FeSODs, most of which are predicted to be chloroplast targeted, and is further dived into two subclades: A1 and A2. Subclade A2 consists of homologues of StFeSOD3, all of them containing a characteristic conserved sequence of 16 amino acids ([RW]A[QE][AS][FL]VNLGEPKIP[VI]A) after the Mn-Fe C-terminal domain. Interestingly, all of the sequences in A2 are predicted to be chloroplast targeted apart from the phylogenetically closest StFeSOD3 and tomato FeSOD SlSOD7 (Solyc02g021140.3.1), which are predicted by TargetP2 to be mitochondrial. However, since the subcellular localization of StFeSOD3 is ambiguous (Table 2), this issue is further considered in the Discussion section. Subclade A1 consists of all the remaining StFeSODs and their homologues including sequences with the E/D rich C-term region associated with PEP, like the Arabidopsis PAP9 (At5g51100.1-*A. thaliana*) and StFeSOD2 sequences. The phylogenetic proximity of SODs associated to PEP and SODs that are not PEP associated indicates that apart the characteristic C-term extension other sequence features are conserved in PEP SODs. All of the sequences in subclade A1 are predicted to be chloroplast targeted apart the potato FeSOD4, predicted to be localized in the cytosol (Figure 6, Table 2).

Based on the phylogenetic tree for CuZnSODs, it can be observed that the differences among the sequences belonging to this SOD class, at least sequences which are homologues to StCuZnSODs, are much less prominent compared to Mn-FeSODs. Each of the StCuZnSODs forms a clade with closest homologues from other plants (Figure 6B). This is not surprising given the high sequence conservation of CuZnSODs among different kingdoms (Figure 5A). The clustering of sequences appears to be related to their subcellular localization—the three clusters correspond to CuZnSOD sequences from cytosol, peroxisome and chloroplast (Figure 6B and Table 2).

In general, all StSODs clustered closely to homologues from the phylogenetically closest plant species—tomato (Figure 6A,B).

### 3.7. Expression Profiles of the StSOD Genes in Response to Elevated Temperatures and SA Application

**Figure 7:**
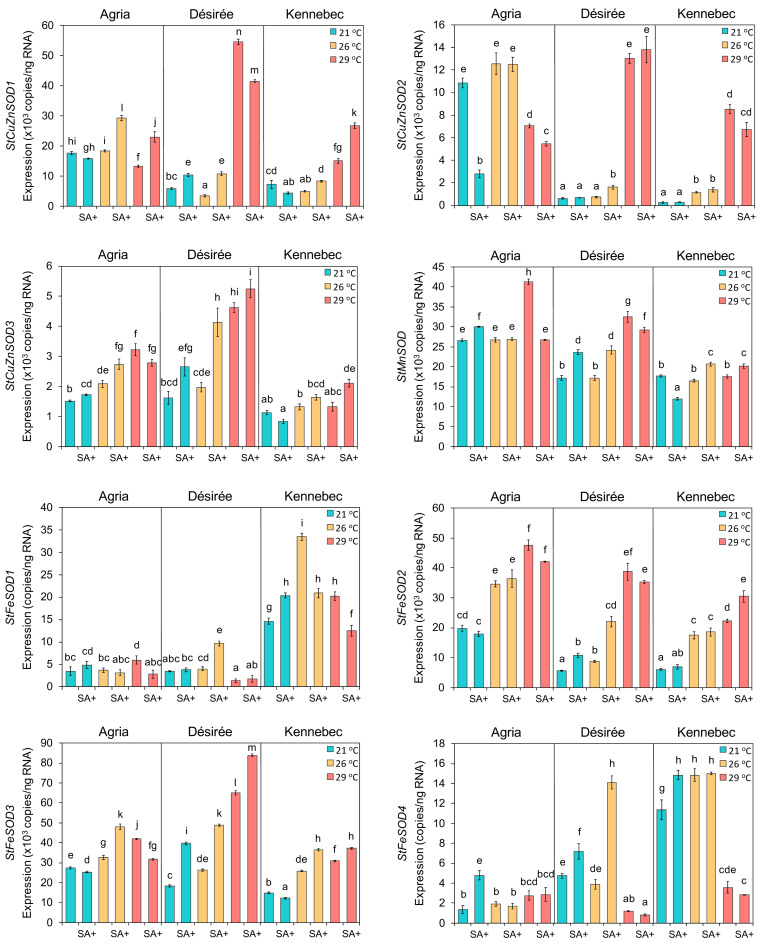
Expression profiles of potato *StSOD* genes under different temperature conditions and exogenous SA application. Real-time PCR was used both to validate the presence of investigated genes and for quantitative analysis of their expression under control (21 °C) and elevated temperatures (26 and 29 °C) with SA application (10^−5^ M) or without. The analysis was conducted on three unrelated potato cultivars (Agria, Désirée, and Kennebec) grown in vitro. The scale bar represents absolute normalized expression values. The data were shown as mean values ± S.D of the three biological replicates. “SA+” indicates exogenous salicylic acid application. The different letters on bars indicate significant differences at a confidence level of *p* < 0.05.

To evaluate the response of *StSOD* genes to elevated temperatures and SA treatment, we have analyzed the expression profiles of these genes in leaves of three potato cultivars (Agria, Désirée, and Kennebec) under three temperature treatments (21 °C, 26 °C, and 29 °C) with or without SA application. Absolute rather than relative quantification by qRT-PCR was used to allow the comparison of the expression levels between different *StSOD*s. In the cases were more gene models (splice variants) were present, the primers were designed to amplify all variants. While all eight *StSOD* transcripts were detected in all three cultivars and under all treatments (Figure 7), the expression levels *StFeSOD1* and *StFeSOD4* were 3–4 orders of magnitude lower as compared to other *StSOD*s, with particularly low expression in Agria and Désirée cultivars. Thus, these two isoforms can be considered minor isoforms.

Regarding the effects of elevated temperature and SA application, no general trends applicable to all *StSOD*s from all cultivars were observed, but some regularities were noted (Figure 7). Mildly elevated temperature of 26 °C did not affect the expression of *StCuZnSODs* and *StMnSOD* in any of the cultivars, except for subtle induction of *StCuZnSOD3* in Agria as compared to control plants. In all examined cultivars, *StFeSOD2* and *StFeSOD3* were up-regulated under 26 °C, while minor isoforms *StFeSOD1* and *StFeSOD4* were up-regulated only in cv. Kennebec with transcript level of *StFeSOD1* increased by 2.3-fold. Treatment of 29 °C induced the expression of all *StSOD*s, except minor isoforms *StFeSOD1* and *StFeSOD4,* in cv. Désirée as compared to the control treatment. The highest differences were observed for *StCuZnSOD1* and *StCuZnSOD2*, which showed 10- and 21-fold higher expression levels at 29 °C than control, respectively. Similar induction of *StCuZnSOD1*, *StCuZnSOD2*, *StFeSOD1*, *StFeSOD2* and *StFeSOD3* was seen in cv. Kennebec in response to 29 °C. In this potato cultivar, the expression level of *StCuZnSOD2* was changed as much as 33-fold when cultivated at 29 °C as compared to control plants. In cv. Agria, however, growth at 29 °C did not cause any major changes in *StSOD*s expression. In this cultivar, the expression levels of *StCuZnSOD3*, *StMnSOD*, *StFeSOD1* and *StFeSOD3* slightly increased (<2.4 fold), while the expression of *StCuZnSOD1* and *StCuZnSOD2* slightly decreased (<1.5 fold) in response to 29 °C, as compared to control.

Modulation of *StSOD*s expression by exogenous SA application in different potato cultivars exposed to three temperature treatments was in most cases subtle. At control temperature of 21 °C, SA had very little effect on *StSOD*s expression, with the exception of a 4-fold down-regulation of *StCuZnSOD2* in cv. Agria. In plants grown at 26 °C, consistent up-regulation of all *StSOD*s in response to SA treatment, as compared to plants grown at the same temperature without SA, was observed only in cv. Désirée, where greatest up-regulation of 3.6-fold was recorded for *StFeSOD4.* In response to SA treatment at 29 °C, almost all *StSOD*s in cv. Agria were slightly down-regulated in comparison to plants grown without SA, with exceptions of *StCuZnSOD1* and *StFeSOD4,* which were up-regulated or unchanged, respectively. Differences in the *StSOD*s expression in cv. Désirée cultivated at 29 °C with or without SA treatment were subtle, and the same is true for cv. Kennebec.

## 4. Discussion

### 4.1. StSOD Gene Family

Comprehensive genome-wide identification and characterization of SOD family members has been conducted in almost all major crops (Table 4), but such data were not available for potato until now. Hereby we present an in depth in silico study of the three *CuZnSOD*s, named *StCuZnSOD1*, *StCuZnSOD2* and *StCuZnSOD3* in the potato genome, along with five members of the Mn-FeSOD class. Sequence Soltu.DM.06G011380 is very likely a single potato *StMnSOD*, whereas others are *FeSOD*s, termed *StFeSOD1* through *StFeSOD4*. Two SOD-like sequences annotated as SODs in DM v6.1 (Soltu.DM.10G011570 and Soltu.DM.06G013120) encode short (~100 aa) proteins, so they can be considered as pseudogenes. Cucumber gene *CsFSD3*, even though it codes for a fairly large 377 aa protein, is also considered as pseudogene because it is not expressed in any organ or under any conditions [61]. Copper chaperone CCS, which is required for the activation of CuZnSODs [62] was also identified (Soltu.DM.08G026370). Considering only papers published since 2015. (Thus probably relying on complete genomic data obtained by state-of-the-art technologies), it seems that a family of 7–9 *SOD* isoforms, with at least one *MnSOD* is typical for most species regardless of genome size (Table 4). Few exceptions with more *SOD*s include banana [63], as well as *Triticum aestivum*, but in the latter case 26 *SOD* isoforms were found in three sub-genomes (A, B and D) of this hexaploid species [64].

### 4.2. Tandem Duplication of the FeSOD Genes Is a Characteristic of Solanum Species

*StSOD* genes are located on five out of 12 potato chromosomes (Table 1), with two genes on chromosome 3 and three genes on chromosome 6. In other plant species *SOD* genes are also scattered on different chromosomes (Table 4). Two physically close genes, *StFeSOD1* and *StFeSOD4*, separated by only 1566 bp on chromosome 6, are 80.77% identical at the protein level and so by definition they represent tandemly duplicated genes [56]. In addition, *StFeSOD1* is 71.91% identical to *StFeSOD2*, but since these genes are on different chromosomes, they can be considered as segmental duplications. Different types of gene duplications are major way for the expansion of gene families, which is often followed by functional divergences of the duplicated genes. However, segmental, rather than tandem duplications have been found for SODs from different plant species. For example, segmental duplications were reported for *OsCSD2* and *OsCSD3* in rice, where both genes preserved their function [65], for *SbSOD2* and *SbSOD5* in *S. bicolor* [66], *GrMSD1* and *GrMSD2* in *G. raimondii* [67], *AtMSD1* and *AtMSD2* as well as *AtFSD1* and *AtFSD2* in *A. thaliana* [67], and *SlSOD5* (Solyc06g048410.2) and *SlSOD6* (Solyc03g095180.2) in tomato [68]. None of the abovementioned species, except tomato, features tandem duplications of *SOD* genes. In tomato, just like in potato, two *FeSOD*s—*SlSOD5* and *SlSOD8* (Solyc06g048420.1) are reported to be tandemly duplicated and in both genomes they are on chromosome 6 [68]. So, both in potato and in tomato one tandem duplication on chromosome 6 (*StFeSOD1/4* and *SlSOD5/8*) and one segmental duplication (*StFeSOD1/2* and *SlSOD5/6*) on chromosomes 6 and 3 were found. Relations among duplicated potato and tomato genes are clear from phylogenetic tree as well (Figure 6A). However, sequence *SlSOD8* was not included in our phylogenetic study, because different versions of this gene, Solyc06g048420.1 and Solyc06g048420.2, code for proteins of 160 and 109 residues respectively, making it unclear whether this is functional gene or a pseudogene. Based on sequence similarities which are greater between tandem pairs than between segmental duplicates in both species, and on the fact that in both species the gene on chromosome 3 does not have its tandem pair [68], it can be concluded that segmental duplication probably preceded tandem duplication event. If so, then *StFeSOD1* in potato and *SlSOD5* in tomato are ancestral genes that first gave rise to copies on chromosome 3 and then were locally duplicated. In any case, tandem duplication of *FeSOD* gene is a relatively recent event characteristic either for the genus *Solanum* or the Solenaceae family.

### 4.3. Gene Models of StSODs

*StSOD* genes have variable number of introns, ranging from 4 to 9 (Figure 1), which is comparable to the number of introns found in *SOD* genes of other species (Table 4). Even genes that are closely related, such as tandem pair *StFeSOD1* and *StFeSOD4*, have quite different exon/intron arrangements (Figure 1). The differences in exon–intron structure of duplicated genes may be accomplished by one of the three main mechanisms: exon/intron, gain/loss, exonization/pseudoexonization, and insertion/deletion [69].

As can be seen in Table 1 and Figure 1, five out of eight *StSOD*s are represented with multiple gene models, where *StFeSOD1* and *StFeSOD4* have as many as six models each. Since protein-coding genes were annotated using full-length cDNAs [24], it is safe to say that these are actually different splice variants generated by alternative splicing. Alternative splicing is involved in the regulation of *SOD* gene expression [70] and has been experimentally proven in rice [71]. In addition to alternative splicing, alternative transcription start sites and alternative polyadenylation has also been reported for *SOD* genes in banana [63]. Such transcripts would share an identical ORF but have different UTRs. This is the case with *StCuZnSOD2*, where its two gene models, Soltu.DM.11G020830.1 and Soltu.DM.11G020830.2, differ in the length of 3′-UTR (Figure 1). It appears that the criterion used for establishing which sequences are representative in Spud DB DM v6.1 is sequence length; in other words, the gene model encoding the longest protein sequence is used as the representative gene model without taking into account structural features of the encoded protein. As discussed below, in some cases this default choice might not be the best choice.

Considering previously discussed tandem and segmental duplications, it is worth noting that duplicated genes, *StFeSOD1*, *StFeSOD2* and *StFeSOD4*, have significantly more splice variants in comparison to other StSOD isoforms (Table 1 and Figure 1). It is tempting to speculate that having three copies of a gene allows for splicing flexibility as a way for further divergence and molecular innovations.

It should be noted that StSOD gene models presented here are not allelic isoforms, since they are derived from a genome of a doubled monoploid clone [24], meaning that the actual molecular variety of these enzymes in potato cultivars, which are highly heterozygous autotetraploids (2n = 4x = 48), is probably even greater.

### 4.4. Structural Features, Subcellular Localization and Phylogenetic Relations of StCuZnSODs

Eukaryotic CuZnSODs are highly conserved proteins regarding their primary structure, position of the key residues, domain organization and tertiary and quaternary structure [72]. StCuZnSODs feature all characteristics of eukaryotic CuZnSODs, including PF00080.22 domain (Figure 3) with conserved residues involved in metal ion binding, dimerization and disulfide bridging (Figure 4A). StCuZnSODs form a typical Greek key scaffold, consisting of a β-barrel composed of eight antiparallel β-strands (Figure 4B).

CuZnSODs are commonly active as homodimers, while chloroplastic CuZnSODs are homotetrameric [5,72,73]. However, native PAGE assays with isoform-selective inhibitors or in combination with immunoblotting revealed at least 6 (and possibly 7, depending on the cultivar and growth conditions) CuZnSOD activity bands in potato [74]. As discussed later, three StCuZnSOD proteins are probably targeted to different cellular compartments, so different subunits should not combine in vivo, but various subunit combinations are possible in the leaf extracts. In the case of *StCuZnSOD*s, alternative splicing as a source of protein variety is not an option, since *StCuZnSOD1* and *StCuZnSOD3* have only one gene model, while the only difference between two *StCuZnSOD2* gene models is polyadenylation site (Figure 1). Other feasible explanations as to why potato cultivars have more CuZnSOD activity bands than genes, include possible post-translational modifications [75] and allelic polymorphism of the isoforms.

Thermostability is another property of enzymes that should be considered when investigating their expression and function under elevated temperatures. Several lines of evidence suggest that CuZnSODs in general, including StCuZnSODs, are thermostable enzymes, expected to perform well during the HS. Average aliphatic index for representative StCuZnSODs is 85.59 (Table 1), which is considerably higher than that of StFeSODs (76.35) but lower than the index of StMnSOD (91.14). The aliphatic index is the relative volume of a protein occupied by aliphatic side chains and may be considered as a positive factor for the increase of thermostability of globular proteins [57]. Furthermore, CuZnSODs, unlike other SODs, feature stable Greek key scaffold which supports active site and dimer formation, and is further reinforced by a disulfide bond (Figure 4) [72]. This disulfide bond stabilizes both the subunit fold and the dimer interface and affects enzyme activity [62,72]. Cu chaperone Cu-CCS not only provides Cu for the active site of the enzyme but facilitates disulfide formation as well [62].

**Figure 8:**
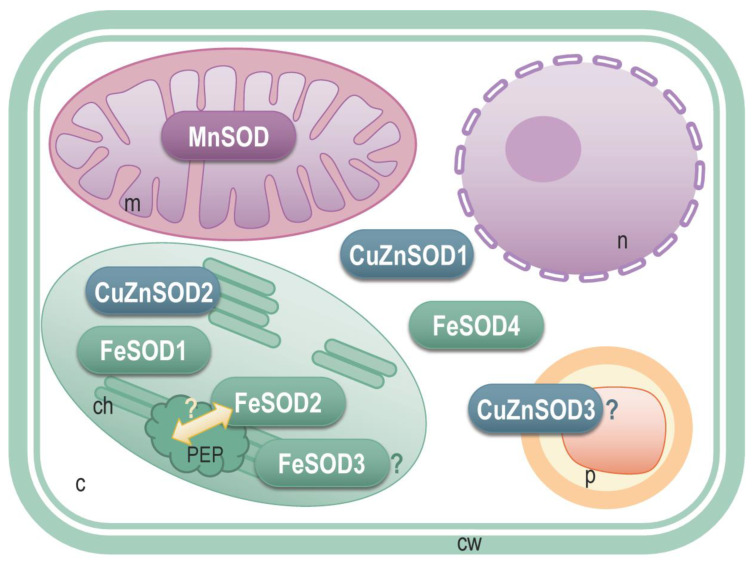
Proposed subcellular localization of StSODs. Prefix “St” is omitted from the potato SOD isoform names for simplicity. C—cytosol; m—mitochondrion; ch—chloroplast; p—peroxisome; n—nucleus; cw—cell wall; PEP—plastid-encoded RNA polymerase. Inconclusive localizations of certain isoforms as well as unproven interactions are indicated by a question mark.

Plant CuZnSODs are found in different cellular compartments, including cytosol, plastids, peroxisomes and possibly extracellular space [5,73]. All four servers inquired for subcellular targeting (DeepLoc, CELLO, TargetP2 and Light Attention) indisputably located StCuZnSOD1 in the cytosol and StCuZnSOD2 in the plastids (Table 2 and Figure 3 and Figure 8). Subcellular localization of StCuZnSOD3, however, is inconclusive. According to DeepLoc-1.0 [31], this sequence is targeted to peroxisomes (Table 2) with likelihood of 0.495, which is higher than cytoplasmic localization likelihood (0.396), whereas other possible localizations are very unlikely. Light Attention [33] also locates StCuZnSOD3 to the peroxisomes, but CELLO does not (Table 2). Programs specifically designed for peroxisomal targeting, like PTS1 Predictor [35,36] or plant-specific PredPlantPTS1 [37] do not predict that StCuZnSOD3 is targeted to peroxisomes. Since most of the peroxisomal proteins possess a peroxisome targeting signal type 1 (PTS1) consisting of a C-terminal tripeptide, the SSV> tripeptide (where “>” is C-terminal end) found in StCuZnSOD3 was compared to PTS1 signals from known peroxisomal proteins [76]. It turned out that SSV> does not belong to so-called canonical plant PTS1 tripeptides, [SA][RK][LMI]>, which confer strong peroxisome targeting efficiency, and not even to weak non-canonical PTS1 tripeptides characterized with one non-canonical amino acid residue at one of these tree positions (x[RK][LMI]>, [SA]y[LMI]> or [SA][RK]z>) [76]. However, SSV> can be found among all known 35 different PTS1 signals that are functional in plants (represented by: [SAPCFVGTLKIQ][RKNMSLHGETFPQCYDA][LMIVYF]>) [76] but its efficiency is yet to be determined. Alternative N-terminal PTS2 motif ([RK][LVIQ]x2[LVIHQ][LSGAK]x[HQ][LAF]) [76] is not found in StCuZnSOD3. Many plants have at least one SOD (whether CuZnSOD, MnSOD, FeSOD or some combination of the isoforms) located in the matrix and/or membrane of the peroxisomes [77], so having at least one peroxisomal SOD must have some physiological advantage.

CuZnSODs share no sequence similarity to Mn-FeSODs and they have probably evolved independently from mutually related FeSODs and MnSODs [5,73], so their relations are presented as two independent phylogenetic trees (Figure 6). In some studies, all *SOD* genes are presented by the same phylogenetic tree, but in these cases CuZnSODs are separated from Mn-FeSODs with high bootstrap values, indicating again their separate evolution [63,67]. Even though CuZnSODs, and specifically plant CuZnSODs, share high degree of homology (Figure 4A), there are some features that distinguish chloroplastic from cytosolic enzymes, which separates them into two clusters [66,68,78]. It can be seen that StCuZnSOD1 clusters with other cytosolic enzymes, whereas StCuZnSOD2 is closely related to chloroplastic CuZnSODs (Figure 6B), which supports previously discussed subcellular localizations. Peroxisomal localization of StCuZnSOD3 is supported by clear phylogenetic association with other peroxisomal SODs (Figure 6B).

### 4.5. StMnSOD Is a Mitochondrial Enzyme with Distinguishable Structural Features

*MnSOD*s and *FeSOD*s have apparently evolved from a common ancestral gene [5,72] and are so closely related, that in literature and sequence databases their products are often referred to as Mn-FeSODs. Both types of enzymes are characterized by the same conserved domains: iron/manganese superoxide dismutase, α-hairpin domain (PF00081.24) and iron/manganese superoxide dismutase, C-terminal domain (PF02777.20, Figure 3). Since all tested potato cultivars express one MnSOD, as confirmed by native PAGE inhibition assays and immunoblots [74], the question is which of the *StSOD* genes encodes MnSOD? We have several reasons to believe that sequence Soltu.DM.06G011380.1 encodes StMnSOD, based on subcellular targeting, structural features and phylogenetic relations. First, protein encoded by Soltu.DM.06G011380.1 is targeted to mitochondria, which is confirmed by four different servers (Table 2 and Figure 3). To our best knowledge, only MnSODs are found in plant mitochondria and it is widely accepted that MnSODs are primarily targeted to mitochondria, even though they can also be found in peroxisomes [5,72,73,77].

Second, there are some structural features that distinguish MnSODs from FeSODs analyzed in our study. In the Mn-FeSODs alignment (Figure 5A) it can be seen that most sequences, all of them being FeSODs, contain Q residue (dark blue) involved in hydrogen-bonding with water at the consensus position 132, whereas in Soltu.DM.06G011380.1 and in two other MnSODs (PDB: 2ADQ, *Homo sapiens* and 6BEJ, *Xanthomonas citri*), this Q residue is at position 223. It is important to note that this Q residue, involved in hydrogen-bonding with the water molecule, assumes a very similar position in the active center regardless of its position in the sequence (either in the loop connecting β2 and β3 strands as in Figure 5C, or in the α3 helix as in Figure 5D), but subtle differences between these two positions affecting the redox tuning of the metal ion define whether the enzyme binds Mn or Fe [79]. This also provides a reason why the amino acids interchangeable with the mentioned Q are short-chained amino acids G and A, since amino acids with longer chain would clash with the opposite Q side chain and prohibit its stabilizing effect on the water molecule ligand.

Even more interesting is the fact that, contrary to StFeSODs, the α-hairpin domain of StMnSOD consists of two long α-helices arranged as a hairpin, resembling the structure of the human mitochondrial MnSOD (PDB: 2ADQ) [60] and other eukaryotic MnSOD structures (PDB: 4X9Q, 4E4E, 4C7U to name a few). This feature is not found in all MnSODs because at least some experimentally resolved structures (for example bacterial MnSODs 6M30 from *Staphylococcus equorum* [80] or 6BEJ from *X. citri* [81]) contain a three-helix α-hairpin domain. The number of helices in the hairpin domain appears to be one of the determinants of MnSOD oligomerization state, where MnSOD variants with two α-helix hairpin domain have a preference to form tetramers. This proposition is based on interaction of the α-hairpin domains of diagonally placed SOD monomers in experimentally determined tetrameric MnSODs, where each side of the oligomer is encircled by one of the two 4-helix bundles at opposite ends of the dimer, which acts as a clamp, holding the dimers in place [82]. It should be noted not all MnSODs containing a two-helix hairpin domain form tetramers in solution, even though they crystalize in the tetrameric state [82].

Finally, these slight structural differences between MnSODs and FeSODs are reflected in the phylogenetic tree as well (Figure 6), where StMnSOD is grouped in cluster B with other MnSODs, whereas all StFeSODs are grouped in cluster A, with other FeSODs. As expected, StMnSOD is closely related to MnSODs from other Solenaceae species—tomato, wild tobacco and pepper (Figure 6).

The fact that StMnSOD has no alternative splice variants (Figure 1) probably reflects the requirement for stringent and, as discussed later, pretty constitutive and high expression, because it is the only mitochondrial SOD isoform in potato, and as such is crucial for ROS scavenging in this organelle. StMnSOD is predicted to be thermostable, having the highest aliphatic index of 91.14 of all StSODs (Table 1).

### 4.6. Not All StFeSOD Splice Variants Encode Functional Proteins

StFeSODs share the same conserved domains PF00081 and PF02777 typical for Mn-FeSODs (Figure 3), but some gene models have certain specificities. As explained in the Results section, sequence Soltu.DM.03G013800.4, encoded by *StFeSOD2*, contains an abnormal 24 amino acid-long insert on the N-terminal side (red highlighted in Figure 5A), while Soltu.DM.06G012170.4, encoded by *StFeSOD4*, contains an aberrant C-terminal region over 60 amino acids long which lacks two conserved amino acid residues involved in metal ion binding (red highlighted in Figure 5A). It is no wonder that these two proteins with abnormal regions, which are, as we believe, produced by abnormal splicing, are products of genes *StFeSOD2* and *StFeSOD4—*segmental and tandem duplicates, respectively, of *StFeSOD1* gene. This only confirms our previous notion that gene duplications in *StFeSOD* family provided certain splicing flexibility, which in two cases resulted in proteins with abnormal regions. While Soltu.DM.03G013800.4 could still be a fully functional enzyme, Soltu.DM.06G012170.4 probably is not. However, our conclusion that gene duplications lead to more flexible alternative splicing, based solely on the study of potato SODs, is in a complete disagreement with findings of [83]. According to comprehensive analysis of these authors, duplicated genes have fewer alternative splice forms than single-copy genes, and there is a negative correlation between the mean number of alternative splice forms and the gene family size [83].

Plant FeSODs are primarily targeted to chloroplasts, but can also be found in cytoplasm, as well as in peroxisomes [5,72,73,77] According to all queried servers, StFeSOD1 and StFeSOD2 are plastidic isoforms (Table 2 and Figure 3 and Figure 8). StFeSOD3 is also predicted to be plastidic by DeepLoc and CELLO, but Light Attention and TargetP2 suggest its mitochondrial location, albeit with low probability (Figure 3). StFeSOD3 clusters with other chloroplastic FeSODs in clade A2 (Figure 6A), and only its closest homolog, tomato FeSOD SlSOD7 (Solyc02g021140.3.1), is also predicted to be mitochondrial by TargetP2 (Figure 6A), but ProtComp9.0 server places it in the chloroplasts [68]. Knowing that only primitive eukaryotes may contain mitochondrial FeSODs [5], StFeSOD3 is probably also targeted to chloroplasts. Finally, regarding StFeSOD4, different servers, except TargetP2, suggest different subcellular localizations for each of its gene models (Table 2). In this case we would incline with the cytosolic (“other”) prediction given by TargetP2, because the N-terminal region preceding the Mn-Fe α-hairpin domain is so short, that any signal would have to overlap with the α-hairpin domain, which is highly unlikely (Figure 3). It could be argued that the chloroplast targeting signal, present in the ancestral *StFeSOD1* gene, has been preserved in segmentally duplicated *StFeSOD2*, but lost in tandemly duplicated *StFeSOD4*.

As already mentioned, StFeSOD2 (Soltu.DM.03G013800.1) is characterized by a long negatively charged C-terminal region rich in E/D (magenta highlighted in Figure 5A). This region resembles the E/D rich C-terminal region of SODs associated with the plastid-encoded RNA polymerase (PEP) multimeric enzyme, like the Arabidopsis PAP9 (also presented in Figure 5A alignment, PDB: 7BJK) [59]. PEP is essential for the proper expression of the plastid genome during chloroplast biogenesis and is composed of four plastid-encoded subunits and 12 nuclear-encoded PEP-associated proteins (PAPs) [59]. Unlike the C-term region of the Arabidopsis PAP9, which is most likely disordered because no electron density was observed for residues after G231 [59], the C-terminal region of Soltu.DM.03G013800.1 is predicted by AlphaFold to be in α-helix conformation (Figure 5B). It is worth noting that the AlphaFold model of Arabidopsis PAP9 (https://www.uniprot.org/uniprot/Q9LU64, accessed on 15 November 2021) agrees with the experimentally determined flexible C-term, so this feature differentiates Soltu.DM.03G013800.1 from PAP9. In addition, PAP9 contains a Zn ion in the active site even though it structurally falls with the Mn-FeSODs [59]. Thus, it would be interesting to experimentally characterize potato StFeSOD2.

The proposed subcellular localization of StSOD enzymes is presented in Figure 8. None of the StSODs localized to the nucleus or extracellular space. Extracellular SODs, if present, are commonly considered to be CuZnSODs [5,73], but in *A. thaliana* the only extracellular SOD is an MnSOD, ATMSD2 (AT3G56350) [9], while in *C. sativus* an FeSOD, CsFSD3, was predicted to be extracellular [61]. However, many plant species including *O. sativa* [9], *S. bicolor* [66], *G. raimondii* and *G. arboreum* [67], *S. lycopersicum* [68] and *T. aestivum* [64] do not have any SODs predicted to be apoplastic.

### 4.7. Expression of StSOD Genes in Response to Elevated Temperatures and Exogenous SA

The ideal temperature for the growth and development of the aerial parts of potato plants is generally 20–25 °C, while the optimal temperature range for tuber formation is 15–20 °C [84]. Even though potato is a cool-season crop, there is variation for HS tolerance across potato germplasm [85,86,87]. We have compared the expression of *StSOD*s in three potato cultivars, Agria, Désirée and Kennebec, grown in vitro either at optimal temperature of 21 °C, or at elevated temperatures of 26 and 29 °C. Désirée is considered as relatively thermo-tolerant cultivar [85,86,87], Agria as thermo-sensitive [87], whereas the data for Kennebec are scarce, but it appears to belong to sensitive cultivars [86]. The described temperature treatments were combined with SA treatments (0 or 10^−5^ M SA), because SA can often enhance thermotolerance by modulating the expression and/or activity of antioxidative enzymes [88,89].

All *StSOD*s identified in doubled monoploid potato genome are present and expressed in tested potato cultivars, which are highly heterozygous autotetraploids (Figure 7 and Figure S1). Even though the current expression analysis (Figure 7) cannot be directly correlated to StSOD activities [74], because different cultivars were tested (only cv. Agria is common to both studies), nevertheless the combined expression and activity data suggest that *StSOD*s can be roughly categorized into: (1) *StCuZnSOD*s, which have relatively high gene expression and high activity; (2) *StSOD*s with relatively high gene expression but low activity, including *StMnSOD*, *StFeSOD2* and *StFeSOD3* and (3) minor isoforms with low expression and low activity—*StFeSOD1* and *StFeSOD4*. Since the dominance of *CuZnSOD*s activities seen in potato plants grown in vitro on medium with limited Cu supply was even more pronounced in ex vitro-grown plantlets [74], the observed changes in expression of *StCuZnSOD*s during different treatments should be regarded as major responses, whereas the changes of expression of other *StSOD*s should be considered as fine tuning of the antioxidative defense.

*StCuZnSOD1* is significantly upregulated at 29 °C in thermotolerant Désirée, and to some degree in Kennebec, but not in thermosensitive cultivar Agria (Figure 7). The expression levels of *StCuZnSOD2* and *StCuZnSOD3* are generally lower as compared to *StCuZnSOD1*, but sharp induction of *StCuZnSOD2* in Désirée and Kennebec at 29 °C and moderate induction of *StCuZnSOD3* in Désirée and Agria at 26 and 29 °C suggest that all three StCuZnSODs may play important roles in protecting their respective compartments—cytosol, chloroplasts and peroxisomes—form ROS generated at elevated temperatures.

*StMnSOD* is practically constitutively expressed with only mild induction at 29 °C in Agria and Désirée (Figure 7). The fact that *StMnSOD,* the only mitochondrial SOD in potato, is not induced by elevated temperatures, and that it has relatively low activity as compared to StCuZnSODs [74], suggests that potato susceptibility to HS may be, in part, due to inadequate antioxidative protection of mitochondria under elevated temperatures. However, when potato (cv. Désirée) was transformed with wheat mitochondrial MnSOD, it showed improvements in HS tolerance [13].

It is interesting that even moderately elevated temperature of 26 °C induced the expression of *StFeSOD2* and *StFeSOD3* in all cultivars, as well as minor *StFeSOD1* and *StFeSOD4* isoforms in cv. Kennebec. Since *StFeSOD1, StFeSOD2* and *StFeSOD3* are predicted to be chloroplastic, it seems that these isoforms, and not *StCuZnSOD2* (whose expression did not increase at 26 °C)*,* are involved in the protection of photosynthetic apparatus during mild temperature stress. The expression of both *StFeSOD2* and *StFeSOD3* further increased with increasing temperature in all three cultivars (Figure 7), suggesting their general role in chloroplasts protection during HS. In cv. Kennebec, however, the expression of *StFeSOD1* and *StFeSOD4* declined at 29 °C as compared to 26 °C treatment, while *StFeSOD4* expression decreased even below the control values. Overall, it seems that cv. Kennebec differs from the other two potato cultivars by generally higher expression of minor isoforms *StFeSOD1* and *StFeSOD4*, which probably have protective roles during mild stress, but somewhat lower expression of other *StSODs*. Thus, the physiological responses of cv. Kennebec to elevated temperatures should be further evaluated and compared to other cultivars. The main difference between thermosensitive Agria and thermotolerant Désirée appears to be sharp inducibility of major *StCuZnSOD1* and *StCuZnSOD2* isoforms at 29 °C in Désirée. Just like in potato, most of the *SOD* genes except mitochondrial *MnSOD* were upregulated by heat treatment in rice [65] and cucumber [61]. In banana, almost all *SOD* isoforms were induced at elevated temperatures [63], while in Arabidopsis only nuclear *CuZnSOD* (At5g18100) was induced by HS [65].

The expression of *StSOD*s was generally unaffected by the application of 10^−5^ M SA, except a mild induction of all *StSOD*s genes in Désirée grown at 26 °C as compared to plants grown at the same temperature on media without SA, and a downregulation of *StCuZnSOD2* in Agria grown at 21 °C (Figure 7). In banana, the expression levels of most *MaSOD* genes were up-regulated in response to the SA treatment, but in this case the plants were treated with 10^−4^ M SA [63]. Foliar application of 6 × 10^−4^ M SA increased both total SOD activity and *StSOD* expression of in potato [90], while in *Impatiens walleriana* grown in vitro, SA in the 1–3 × 10^−3^ M range reduced SOD activity in a dose-response manner [91]. SA concentration of 10^−5^ M used in present work was selected based on unpublished results with different potato cultivars and literature data [53], but it is possible that higher concentrations of SA are required for effective regulation of *StSOD*s expression in the studied cultivars. It can also be argued that in thermotolerant Désirée, SA application at 26 °C helps in stress amelioration by induction of *StSODs*, while at higher temperatures other mechanisms by which SA may enhance plants’ thermotolerance are in action. Namely, SA may be directly or indirectly involved in the improvement of thermotolerance in various plant species by: stimulation of Pro synthesis to improve water uptake under HS, enhancement of different antioxidant enzymes activities and/or expression, protection of photosynthetic apparatus by increase of RUBISCO activity, inhibition of D1 protein degradation and other mechanisms, effects on other phytohormones and crosstalk among them, and induction of HSPs expression [7,92].

### 4.8. StCuZnSOD1 Promoter Features Many More Cis-Acting Regulatory Elements Than Other StSOD Promoters and Is the Only One Predicted to Be Regulated by Heat or SA

Gene expression is transcriptionally regulated via the change in the level or activity of TFs that bind to specific cis-acting promoter elements. We have performed in silico analysis of StSOD promoters in order to evaluate the potential for transcriptional regulation of different isoforms (Figure 2). Despite similarities in expression profiles of *StCuZnSOD*s, *StCuZnSOD1* promoter substantially differs from other *StSOD* promoters in terms of the number of identified cis-regulatory elements (Figure 2A). Namely, *StCuZnSOD1* promoter is characterized by numerous, often overlapping cis-elements for binding different TFs, primarily from the ERF, Dof and LBD families, but also Heat stress TFs (HSFs)—major regulators of HS and other stress responses in plants [93] that are not found in other potato *SOD* promoters. There are two partially overlapping HSF cis-elements proximal to the transcription site, which correspond to HSFs involved in response to ROS and chitin (see Tables S2 and S3 for details). As indicated in Figure 2B, the TFs from the ERF, Dof, LBD, MYB and other families, predicted to recognize *StCuZnSOD1* promoter sequences, are implicated in responses to different stresses, phytohormones and developmental signals. Among them, an ERF TF is involved in heat acclimation, while three TFs belonging to Dof, GRAS and MYB families are involved in response to SA (Tables S2 and S3). All this suggests that cytosolic *StCuZnSOD1* is a common component of a number of signaling networks, possibly involved in controlling the level of H_2_O_2_, which is a well-recognized signaling molecule.

Unlike *StCuZnSOD1* promoter, *StCuZnSOD2* promoter features a single MYB binding site, which are also present in *StCuZnSOD1* and *StFeSOD4* promoters. Among other functions, some members of the MYB TF family are implicated in heat tolerance [94,95]. As a matter of fact, many other TF types with cis-elements in *StSOD*s promoters (Figure 2A) can also be related to HS responses: certain members of M-type MADS, bZIP, as well as Dof family play important roles in modulating HS response in plants [95,96], while many of the ERF TFs identified in potato genome are involved in HS response [97]. However, since classification of TFs into families is generally based on their characteristic DNA-binding domains, it is not informative in terms of biological processes in which specific TFs are implicated. Thus, TFs predicted to bind *StSODs* promoters were associated with GO terms for biological processes (Figure 2B and Table S3), revealing that none of them is specifically involved in response to heat. This is in contrast with findings that majority of *SOD* promoters from other investigated species such as banana, tomato or cucumber apparently have more cis-regulatory elements as compared to potato *SOD*s (with a notable exception of *StCuZnSOD1*), featuring not only more HSFs and SA-responsive elements, but different types of elements as well [61,63,68]. Common factor underlying the mentioned reports is the use of highly cited but somewhat outdated PlantCARE server for identification of plant cis-acting regulatory elements [29], whereas we have used an up to date approach based on PlantRegMap [26] and PlantTFDB v5.0 [28] which probe a more diverse motif landscape. In order to control the rate of falsely detected binding sites we used a stringent threshold for assigning a motif. Ultimately this produces quite different results compared to using PlantCARE on the same promoter sequences (see Table S4, where *StSOD* promoters were analyzed using PlantCARE, for a comparison, as well as Table S5, with comparative list of cis-elements found using both platforms). We trust that the accumulated knowledge in the almost two decades that separate PlantCARE from PlantRegMap should not be ignored.

Even though our in silico analysis of *StSOD* promoters (Figure 2 and supplementary materials) cannot fully support expressional data (Figure 7), it clearly demonstrates dependence of in silico promoter analyses on the adopted methods and a requirement for more experimental evidence on the interactions between different TFs and their cis-elements in plants. Finally, regarding the involvement of different StSODs in HS responses, it should be underlined again that StSODs, like other SODs, can probably be regulated not only at transcriptional level, but also post-transcriptionally at the level of alternative splicing [63,70,71] and by post-translational modifications [75]. CuZnSODs are additionally regulated by miRNAs, specifically miR398 [98], activated by copper chaperone CCS [62] and regulated by Cu availability [74,99].

## 5. Conclusions

Herby we present the first detailed insight into the SOD gene/protein family in potato. Exon-intron organization, splice variants, cis-regulatory promoter elements and chromosome localization of the eight functional *StSOD* genes has been described, along with structural features, subcellular localization, and phylogenetic relations of the StSOD proteins.

Investigation of the scope and time of the tandem *FeSOD* duplication event, characteristic for tomato and potato, would require comparative analysis of *FeSODs* in other Solenaceae species, when genomic resources become available. For determination of subcellular localization of StSODs, multiple servers were inquired and compared with phylogenetic and literature data, and yet peroxisomal targeting of StCuZnSOD3 remained inconclusive. Therefore, one of the future prospects is to experimentally determine whether there is a CuZnSOD activity in potato peroxisomes.

Higher induction of all *StCuZnSOD*s, *StFeSOD3* and even *StMnSOD* in thermotolerant Désirée grown at 29 °C as compared to thermosensitive Agria and Kennebec, suggests that thermotolerance in potato might be related to induction of these isoforms. In addition, protection of chloroplasts under mild stress of 26 °C is apparently mediated by increased expression of chloroplastic *StFeSODs*. The application of low concentration of SA caused a mild induction of all *StSOD*s, but only in Désirée grown at 26 °C, suggesting that ameliorating effects of SA during HS described in literature probably also include other mechanisms, as well as crosstalk among different phytohormones. Further spatio-temporal analysis of *StSODs* expression, however, should be refined to distinguish and quantify specific *StFeSODs* splice variants in different tissues and under different conditions.

We believe that our findings will aid future investigations of SODs roles and regulation in potato, particularly in relation to heat stress.

## Figures and Tables

**Table 1:** Description of the potato SOD (*StSOD*) genes and proteins analyzed in this study.

Proposed Name	Gene ID	Chromosomal Localization	Transcript ID	Transcript Length (bp)	Protein Length (aa)	Isoelectric Point (pI)	Instability Index	Aliphatic Index	Molecular Weight (kDa)
*StCuZnSOD1*	Soltu.DM.01G022650	Chr.01: 61,097,981-61,103,305	Soltu.DM.01G022650.1 *	459	152	5.28	32.24	80.20	15.3
*StCuZnSOD2*	Soltu.DM.11G020830	Chr.11: 40,784,322-40,790,930 Chr.11: 40,784,322-40,790,564	Soltu.DM.11G020830.1 * Soltu.DM.11G020830.2	648 645	215 214	6.34 6.34	24.76 24.25	87.95 88.36	22.1 22.1
*StCuZnSOD3*	Soltu.DM.03G010200	Chr.03: 28,151,770-28,156,700	Soltu.DM.03G010200.1 *	498	165	6.78	18.64	88.61	16.8
*StMnSOD*	Soltu.DM.06G011380	Chr.06: 34,521,324-34,524,092	Soltu.DM.06G011380.1 *	687	228	7.13	35.60	91.14	25.3
*StFeSOD1*	Soltu.DM.06G012180	Chr.06: 36,014,174-36,016,425 Chr.06: 36,014,151-36,016,425 Chr.06: 36,014,174-36,016,425 Chr.06: 36,014,174-36,016,425 Chr.06: 36,014,174-36,016,425 Chr.06: 36,014,174-36,016,425	Soltu.DM.06G012180.1 Soltu.DM.06G012180.2 Soltu.DM.06G012180.3 * Soltu.DM.06G012180.4 Soltu.DM.06G012180.5 Soltu.DM.06G012180.6	747 753 759 729 681 654	248 250 252 242 226 217	6.31 6.60 6.60 7.77 5.96 6.52	33.40 33.86 34.62 35.05 35.28 35.44	70.56 70.00 70.99 75.08 74.38 70.74	27.8 28.1 28.3 26.9 25.1 24.1
*StFeSOD2*	Soltu.DM.03G013800	Chr.03: 36,413,200-36,416,935 Chr.03: 36,413,200-36,416,935 Chr.03: 36,413,200-36,416,935 Chr.03: 36,413,200-36,416,935 Chr.03: 36,413,200-36,416,935	Soltu.DM.03G013800.1 Soltu.DM.03G013800.2 Soltu.DM.03G013800.3 Soltu.DM.03G013800.4 * Soltu.DM.03G013800.5	915 891 897 987 840	304 296 298 328 279	5.56 5.49 5.83 5.69 5.20	41.47 41.99 40.95 40.11 42.01	73.26 73.58 72.11 76.19 73.87	34.7 33.7 33.8 37.6 31.9
*StFeSOD3*	Soltu.DM.02G001300	Chr.02: 7,378,597-7,382,073 Chr.02: 7,378,597-7,382,073	Soltu.DM.02G001300.1 * Soltu.DM.02G001300.2	777 753	258 250	6.07 5.96	42.61 43.46	85.04 85.80	29.6 28.5
*StFeSOD4*	Soltu.DM.06G012170	Chr.06: 36,008,330-36,012,608 Chr.06: 36,008,330-36,012,608 Chr.06: 36,008,330-36,012,608 Chr.06: 36,008,330-36,012,608 Chr.06: 36,008,330-36,012,608	Soltu.DM.06G012170.1 Soltu.DM.06G012170.2 Soltu.DM.06G012170.3 Soltu.DM.06G012170.4 * Soltu.DM.06G012170.5	603 540 549 684 513	200 179 182 227 170	5.58 5.81 6.29 7.85 6.12	31.53 33.93 30.48 39.93 35.64	82.00 78.60 76.26 73.17 74.18	22.5 20.2 20.4 25.6 19.1
		Chr.06: 36,008,330-36,012,608	Soltu.DM.06G012170.6	633	210	5.89	31.93	77.71	23.8

Note: * indicates representative sequences; bp, base pair; aa, amino acid.

**Table 2:** Comparative subcellular localization prediction of potato SODs by different tools.

Gene	Protein ID	TargetP2	DeepLoc	CELLO	LA	PTS1 Predictor	PredPlant PTS1
*StCuZnSOD1*	Soltu.DM.01G022650.1	O	C	C	C		
*StCuZnSOD2*	Soltu.DM.11G020830.1 Soltu.DM.11G020830.2	Ch Ch	Ch Ch	Ch Ch	Ch Ch		
*StCuZnSOD3*	Soltu.DM.03G010200.1	O	P	C	P	Not-P	Not-P
*StMnSOD*	Soltu.DM.06G011380.1	Mt	Mt	Mt	Mt		
*StFeSOD1*	Soltu.DM.06G012180.1 Soltu.DM.06G012180.2 Soltu.DM.06G012180.3 Soltu.DM.06G012180.4 Soltu.DM.06G012180.5 Soltu.DM.06G012180.6	Ch Ch Ch Ch Ch Ch	Ch Ch Ch Ch Ch Ch	Ch Ch Ch Ch Ch Ch	Ch Ch Ch Ch Ch Ch		
*StFeSOD2*	Soltu.DM.03G013800.1 Soltu.DM.03G013800.2 Soltu.DM.03G013800.3 Soltu.DM.03G013800.4 Soltu.DM.03G013800.5	Ch Ch Ch Ch Ch	Ch Ch Ch Ch Ch	Ch Ch Ch Ch, C Ch	Ch Ch Ch Ch Mt		
*StFeSOD3*	Soltu.DM.02G001300.1 Soltu.DM.02G001300.2	Mt Mt	Ch Ch	Ch Ch	Mt Mt		
*StFeSOD4*	Soltu.DM.06G012170.1 Soltu.DM.06G012170.2 Soltu.DM.06G012170.3 Soltu.DM.06G012170.4 Soltu.DM.06G012170.5	O O O O O	Mt Mt Mt Mt Mt	Ch, C Ch, C Ch, C, Mt N, Mt Ch, C, Mt	Mt C C Mt Mt		
	Soltu.DM.06G012170.6	O	Mt	Ch	C		

Note: C, cytosolic; Ch, chloroplastic; P, peroxisomal; Mt, mitochondrial; N, nuclear; O, other (because TargetP2 does not assign cytosolic localization).

**Table 3:** Evaluation of StSOD AlphaFold models.

Protein ID	Ramachandran Favored	Ramachandran Outliers	Rotamer Outliers	Clashscore
Soltu.DM.01G022650.1	98.67%	0.00%	0.00%	0.95
Soltu.DM.11G020830.1	98.03%	0.00%	0.00%	0.00
Soltu.DM.03G010200.1	92.02%	2.45%	0.00%	0.00
Soltu.DM.06G011380.1	98.51%	0.00%	0.00%	0.95
Soltu.DM.06G012180.3	98.01%	0.00%	0.00%	0.93
Soltu.DM.03G013800.1	97.24%	0.39%	0.91%	0.49
Soltu.DM.02G001300.1	95.83%	0.00%	0.00%	2.58
Soltu.DM.06G012170.6	97.60%	0.48%	0.57%	2.11

**Table 4:** Overview of the *SOD* gene families in different crops.

Species	Genome Size (Mbp)	*CuZnSOD*	*MnSOD*	*FeSOD*	Total	Chr. num.	Introns num.	References
*Solanum tuberosum*	844	3	1	4	8	5	4–9	Current work
*Arabidopsis thaliana*	125	3	2	3	8	5	5–8	[9,65]
*Oryza sativa*	389	4	1	2	7	6	5–9	[9,65]
*Sorghum bicolor*	730	5 *	1	2	8	6	5–7	[66]
*Gossypium raimondii*	885	5	2	2	9	6+	4–8	[67]
*Gossypium arboreum*	1746	5	2	2	9	6	5–8	[67]
*Cucumis sativus*	367	5	1	3	9	5+	3–8	[61]
*Musa acuminate*	523	6	4	2	12	8	5–8	[63]
* Solanum lycopersicum *	828	4 *	1	4	9	6	4–8	[68]
*Triticum aestivum*	~17,000	17	3	6	26	3 × 3	4–7	[64]

Note: The listed number of CuZnSOD isoforms does not include Cu chaperones, but cases where it is not clear whether Cu chaperones are included are indicated with an asterisks (*). Chr. num. is the number of chromosomes where *SOD* genes are located, where “+” indicates that some *SOD* genes were found on scaffolds. In wheat, *SOD* genes are found on 3 chromosomes of each of the 3 sub-genomes.

## Data Availability

Data are contained within the article and supplementary material.

## Supplementary Materials

The following supporting information can be downloaded at: https://www.mdpi.com/article/10.3390/antiox11030488/s1, Figure S1: Confirmation of amplicon size and primer specificity of studied *StSOD* genes, Figure S2: Alignments of Mn-FeSOD and CuZnSOD proteins used for phylogeny reconstruction, Table S1: PCR primer sequences used in this study for gene expression analysis by qRT-PCR, Table S2: Transcription factor binding sites identified in the promotor regions of *StSODs*, Table S3: Biological process GO terms associated with transcription factors binding promoters of *StSODs*, Table S4: Results of cis-element analysis in the promoter of *StSODs* using the PlantCARE web tool, Table S5: Comparison of results obtained from PlantCARE and PlantTFBD databases, Supplementary Info S6: AlphaFold models of StSOD proteins (PDB files) and Ramachandran diagrams of the respective models.
