# Supplementary figures and images for: In Silico Study of Superoxide Dismutase Gene Family in Potato and Effects of Elevated Temperature and Salicylic Acid on Gene Expression
Source: Antioxidants (Basel). 2022 Feb 28;11(3):488. doi: 10.3390/antiox11030488 (PMC8944489; doi:10.3390/antiox11030488)

General

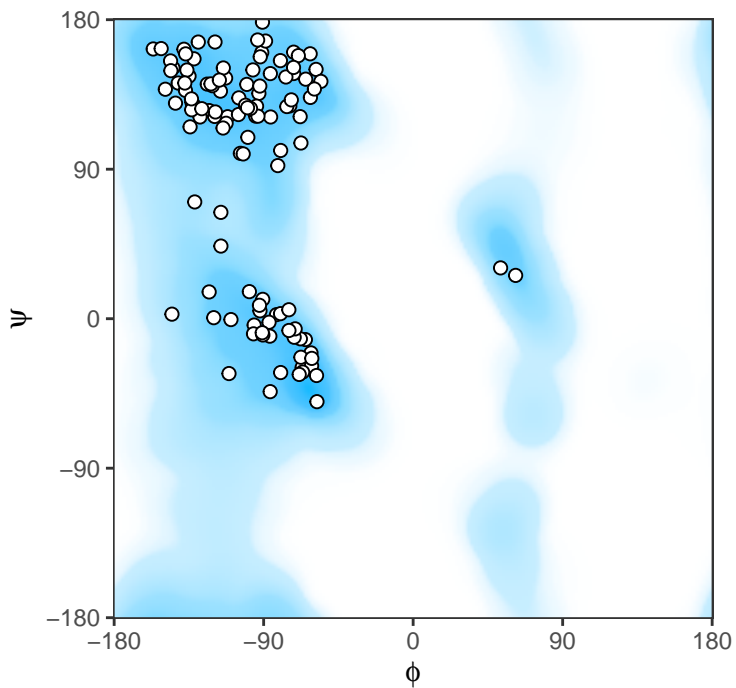

Glycine (Symmetric)

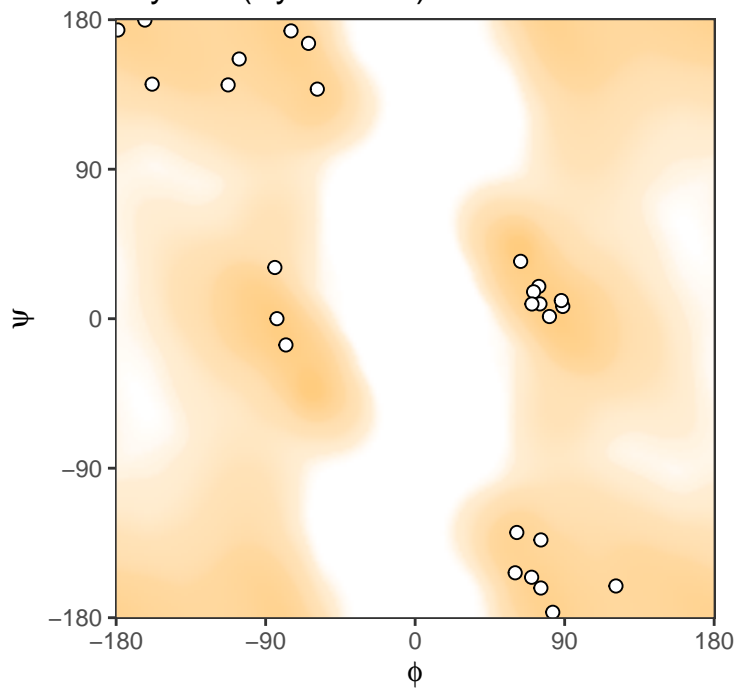

Pre-Proline

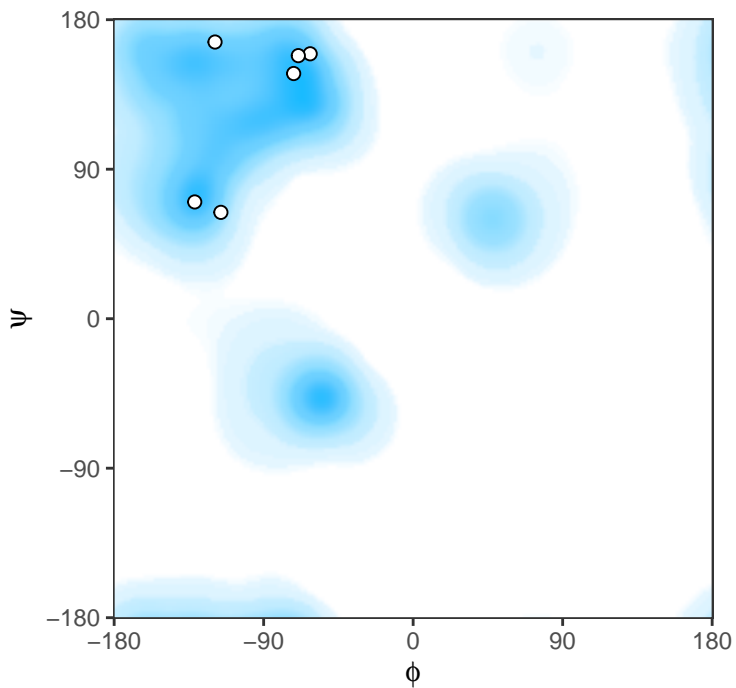

Proline

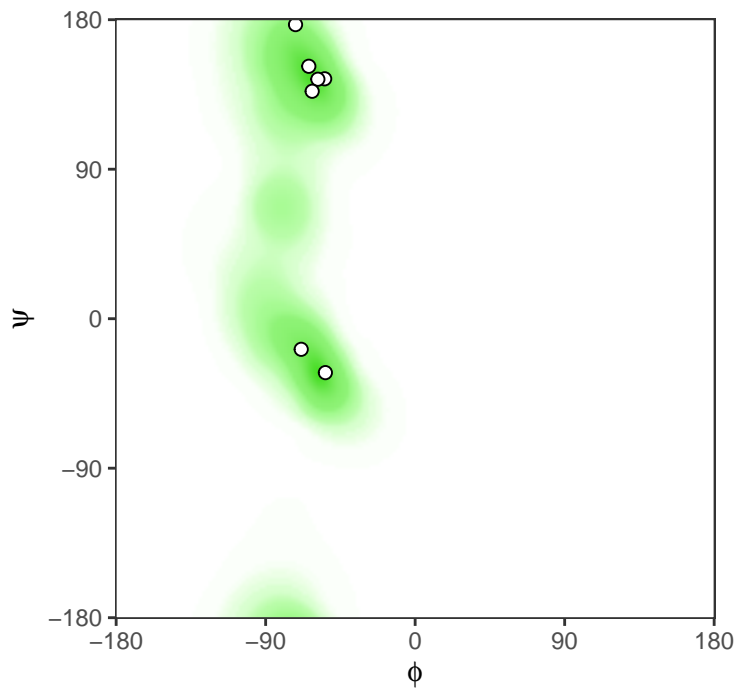

Supplement: Supplementary file 1 [file antioxidants-11-00488-s001.zip › Supplement S7/Soltu.DM.01G022650.1_ramachandran.pdf]

General

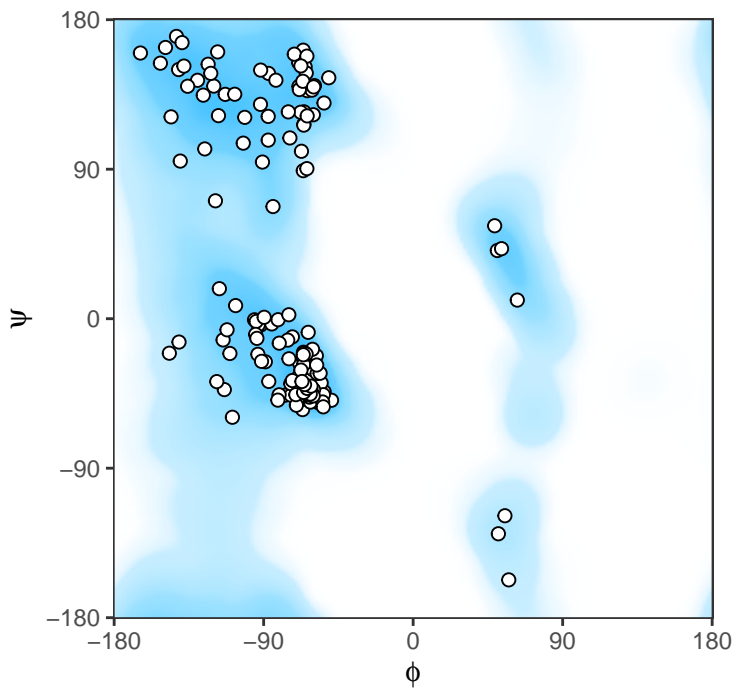

Glycine (Symmetric)

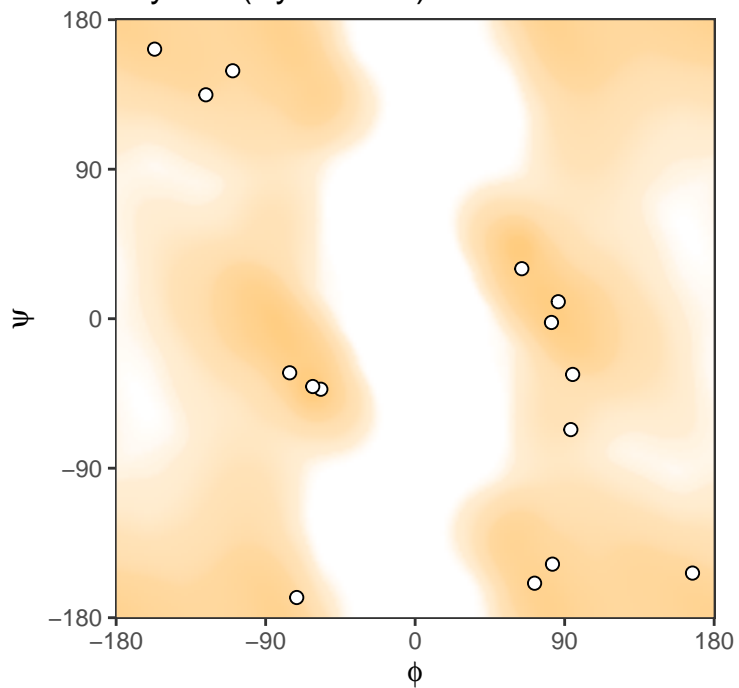

Pre-Proline

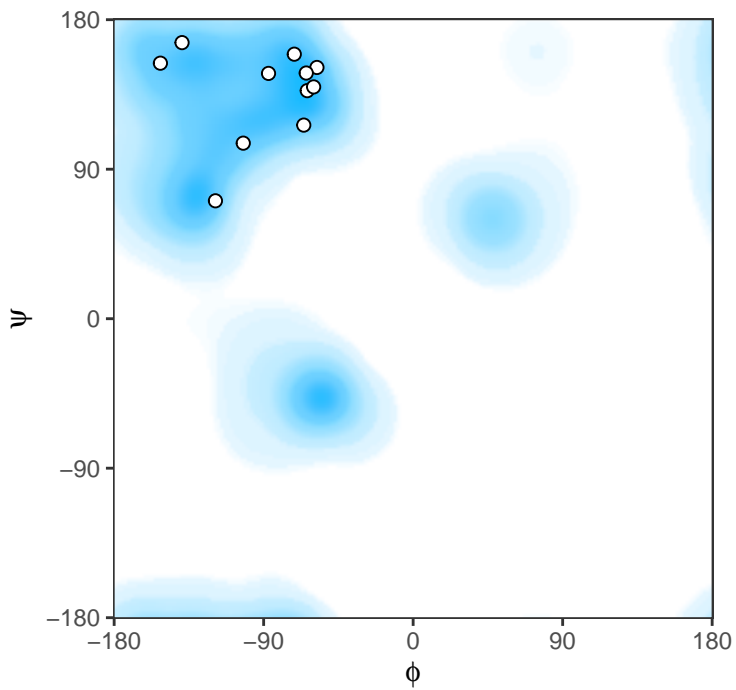

Proline

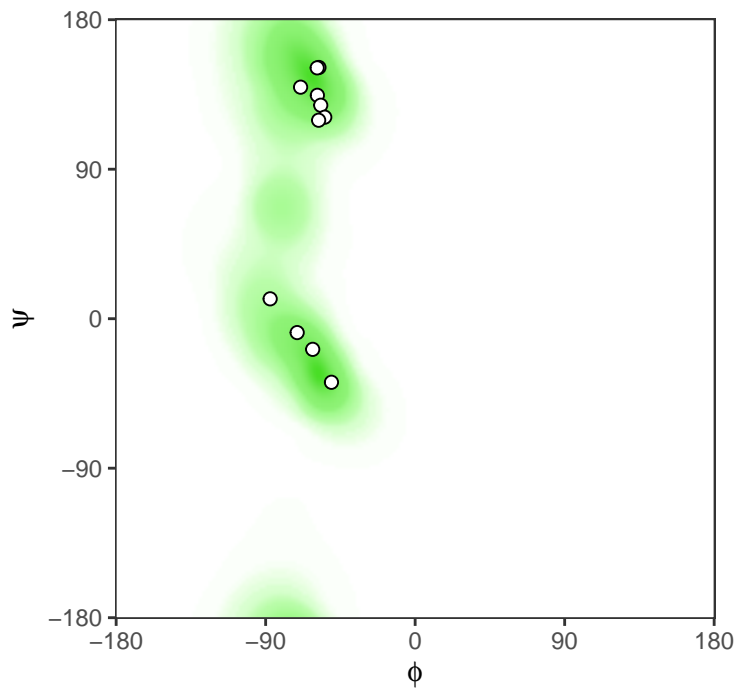

Supplement: Supplementary file 1 [file antioxidants-11-00488-s001.zip › Supplement S7/Soltu.DM.02G001300.1_ramachandran.pdf]

General

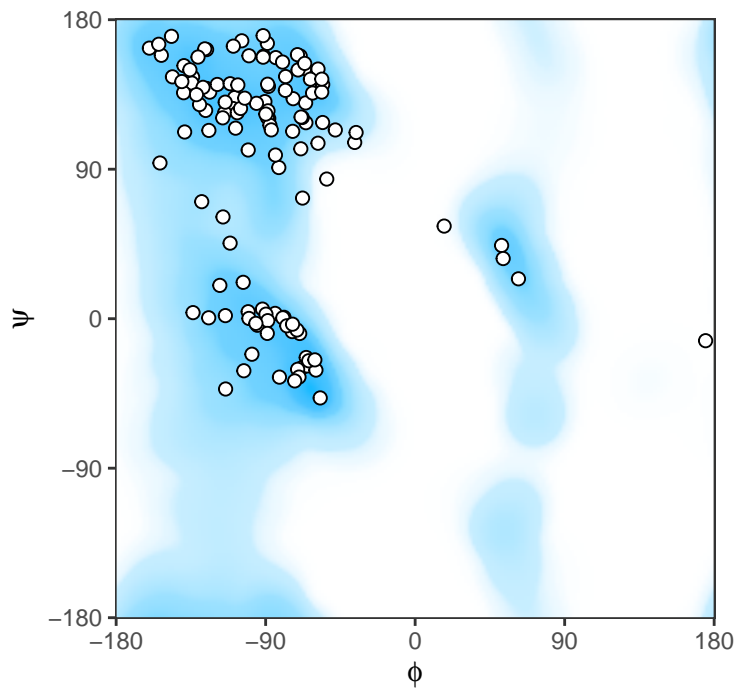

Glycine (Symmetric)

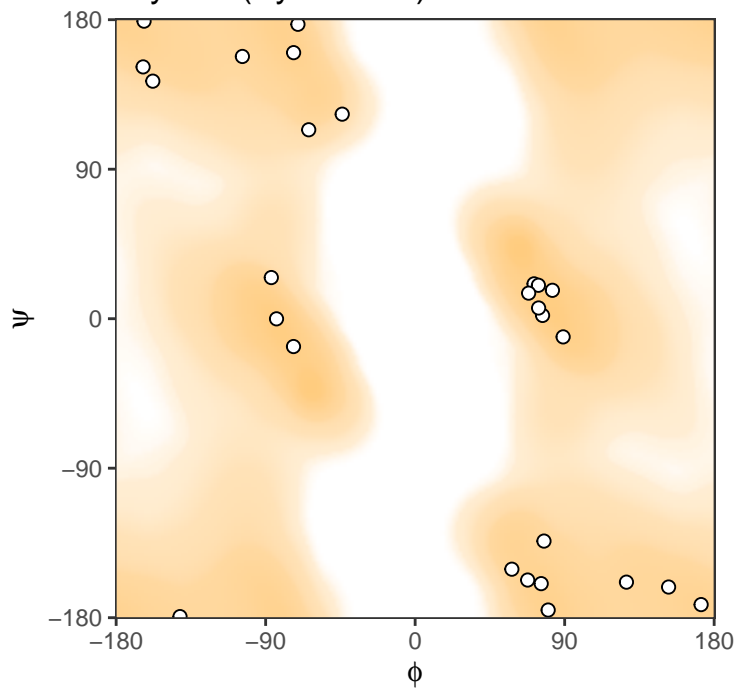

Pre-Proline

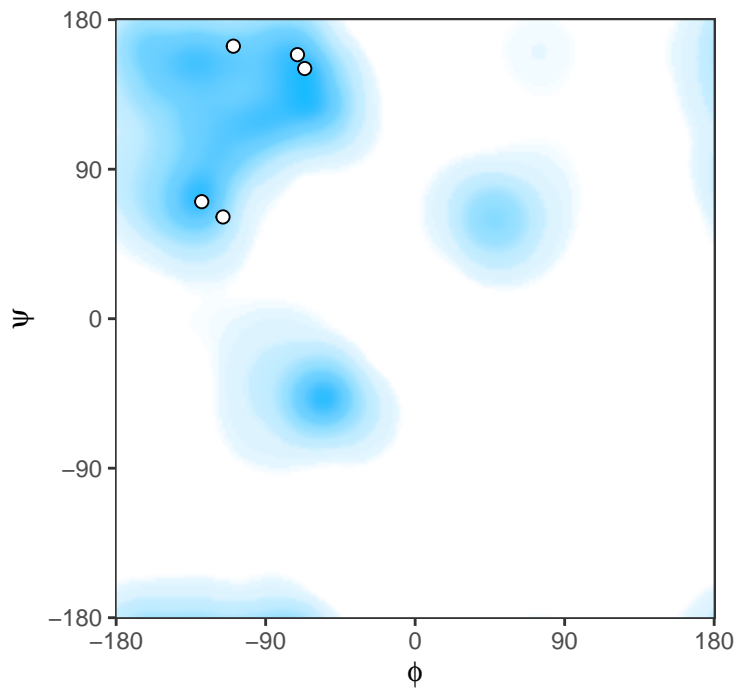

Proline

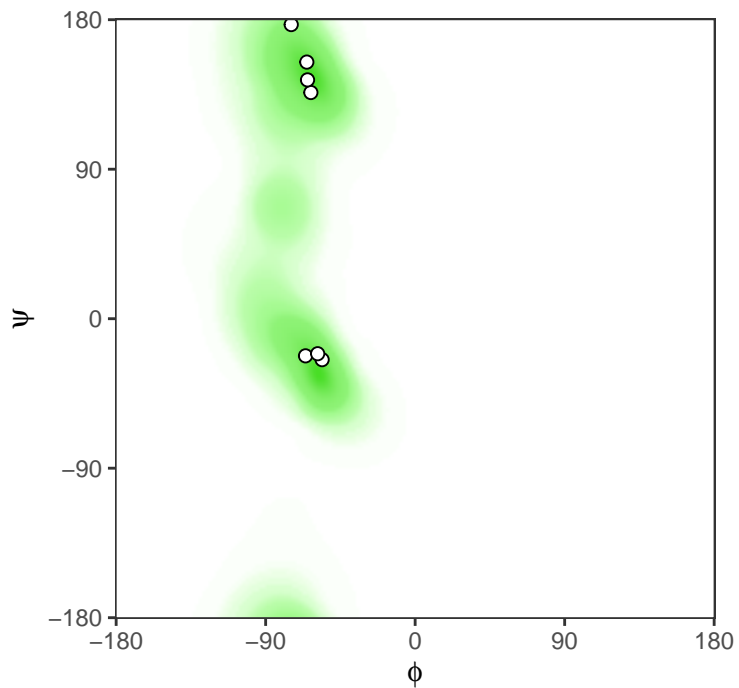

Supplement: Supplementary file 1 [file antioxidants-11-00488-s001.zip › Supplement S7/Soltu.DM.03G010200.1_ramachandran.pdf]

General

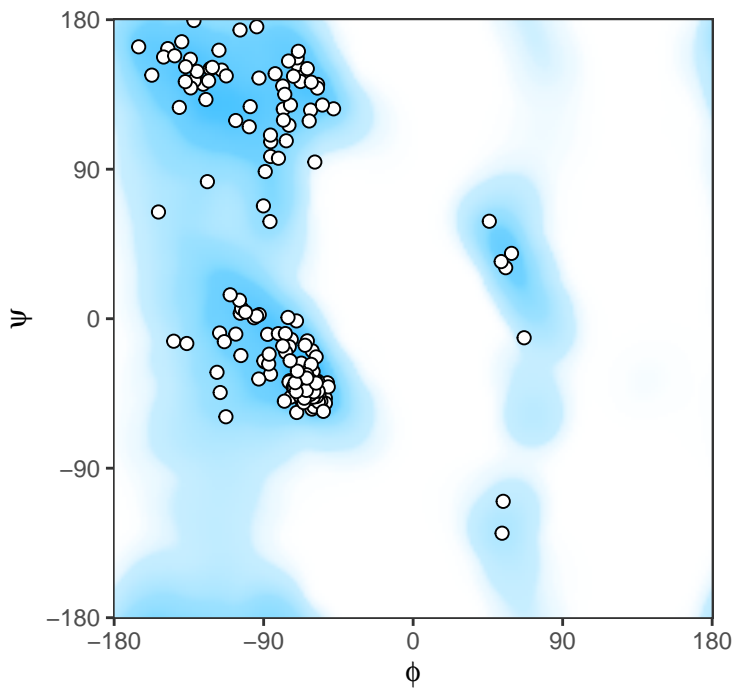

Glycine (Symmetric)

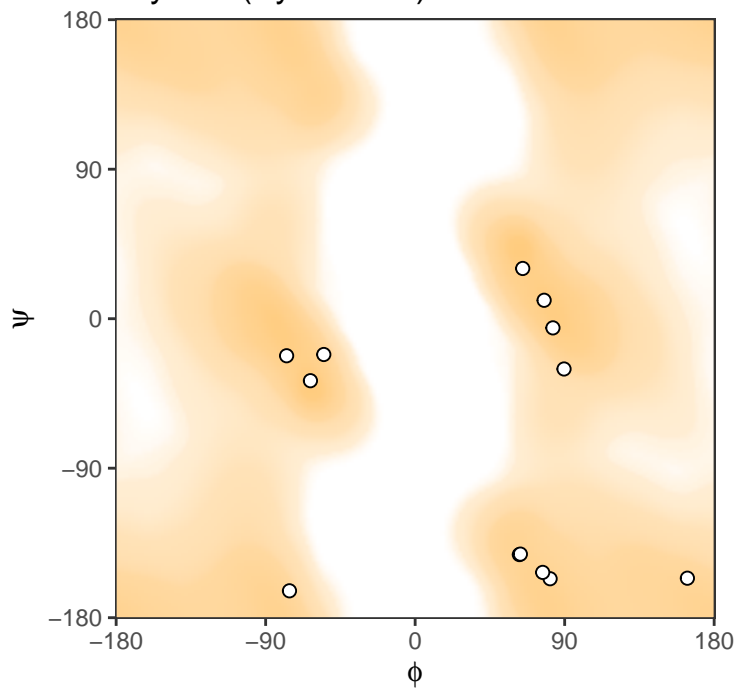

Pre-Proline

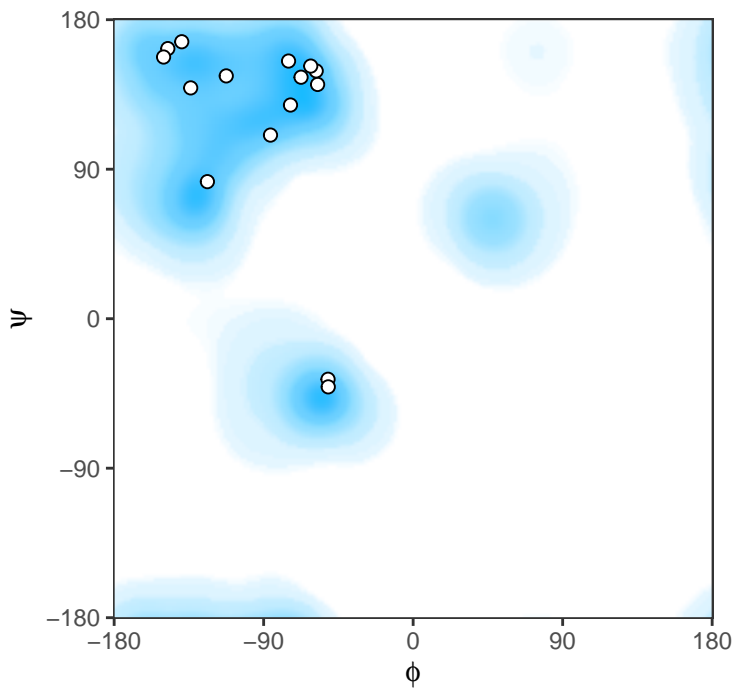

Proline

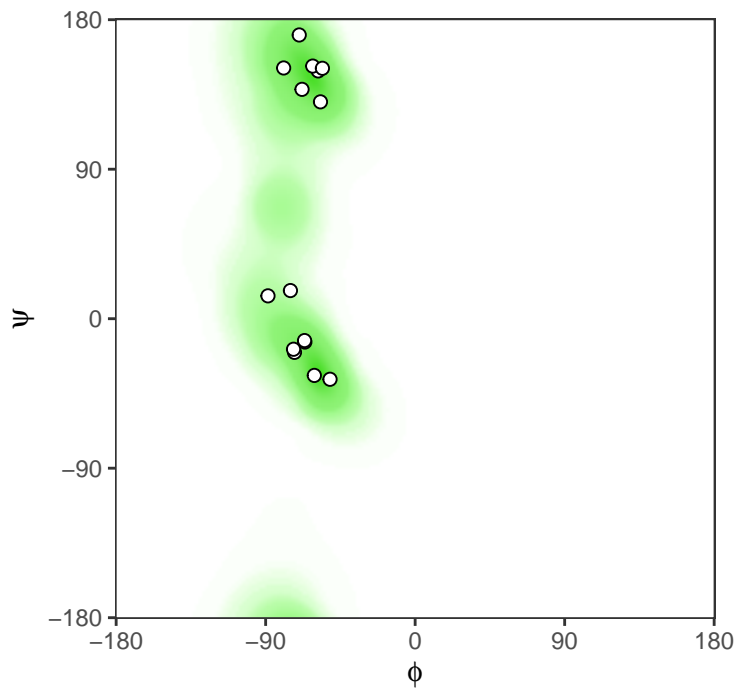

Supplement: Supplementary file 1 [file antioxidants-11-00488-s001.zip › Supplement S7/Soltu.DM.03G013800.1_ramachandran.pdf]

General

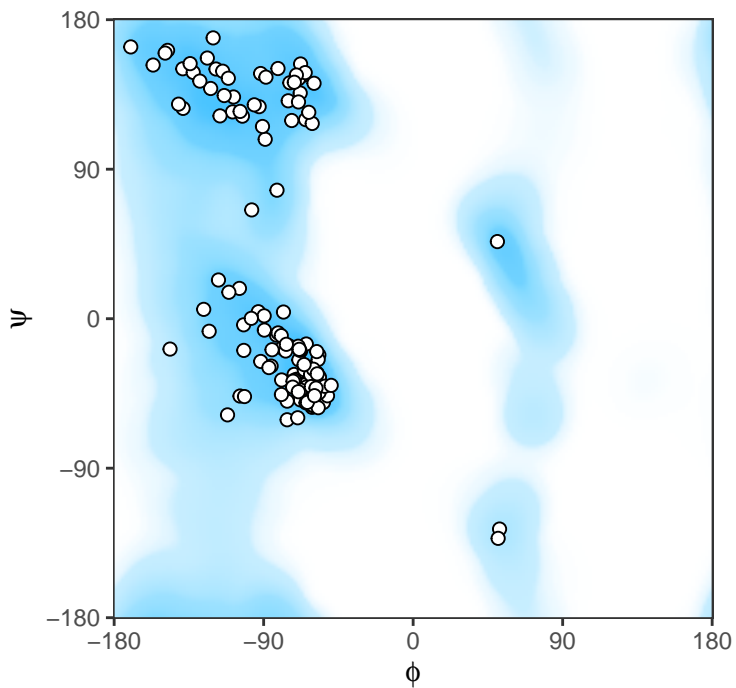

Glycine (Symmetric)

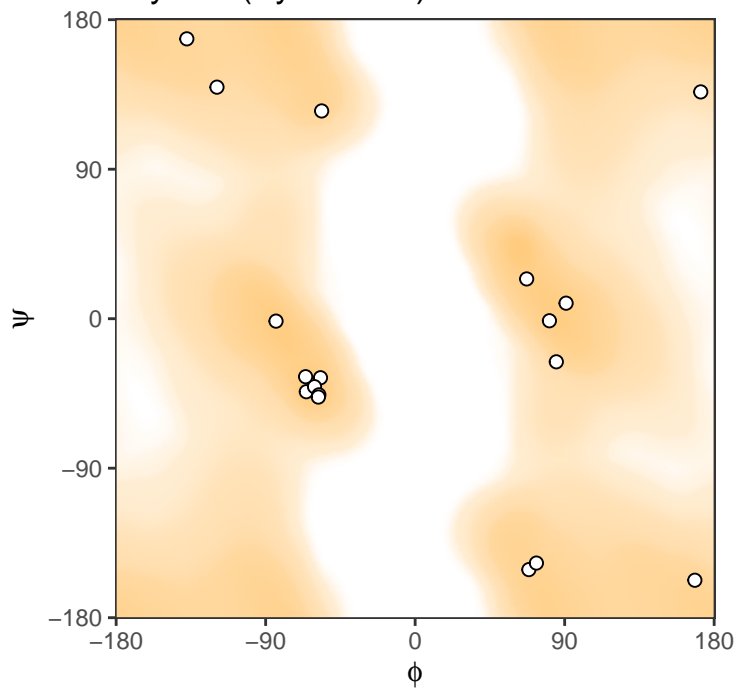

Pre-Proline

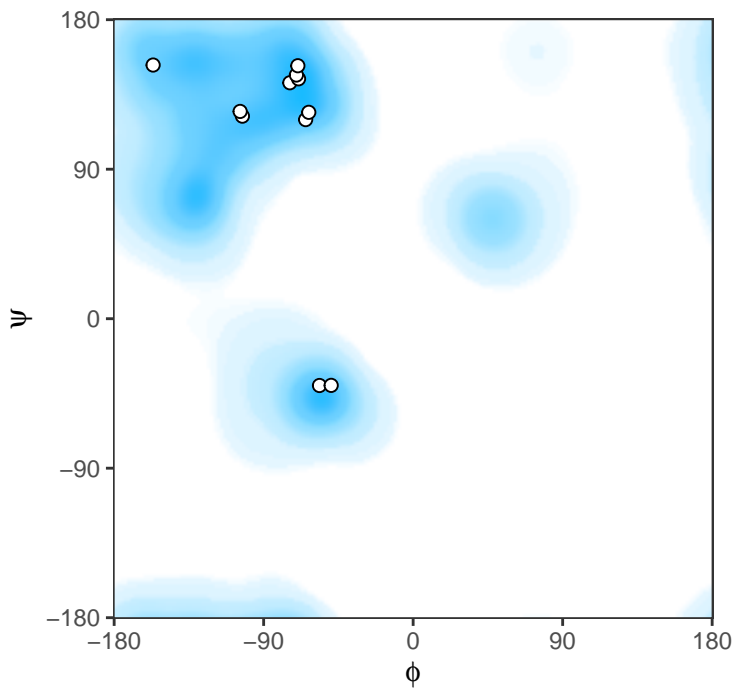

Proline

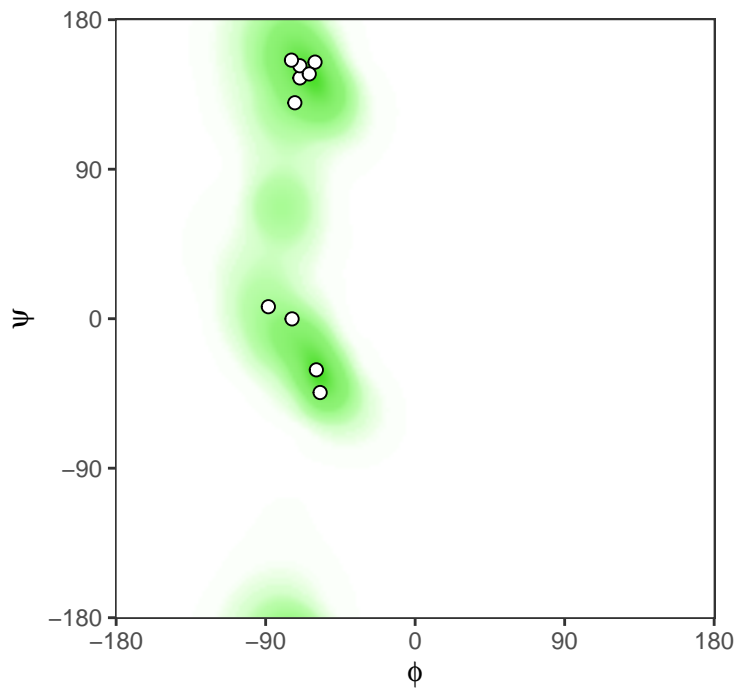

Supplement: Supplementary file 1 [file antioxidants-11-00488-s001.zip › Supplement S7/Soltu.DM.06G011380.1_ramachandran.pdf]

General

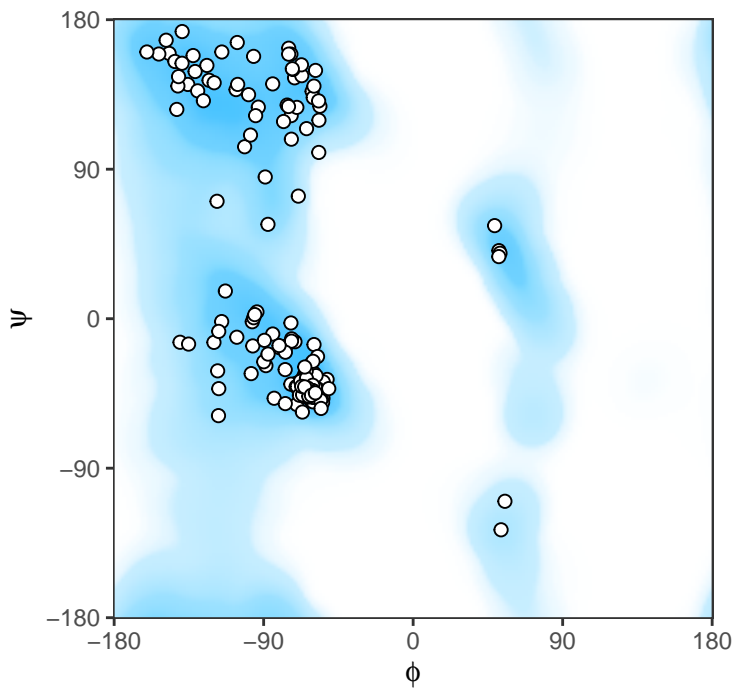

Glycine (Symmetric)

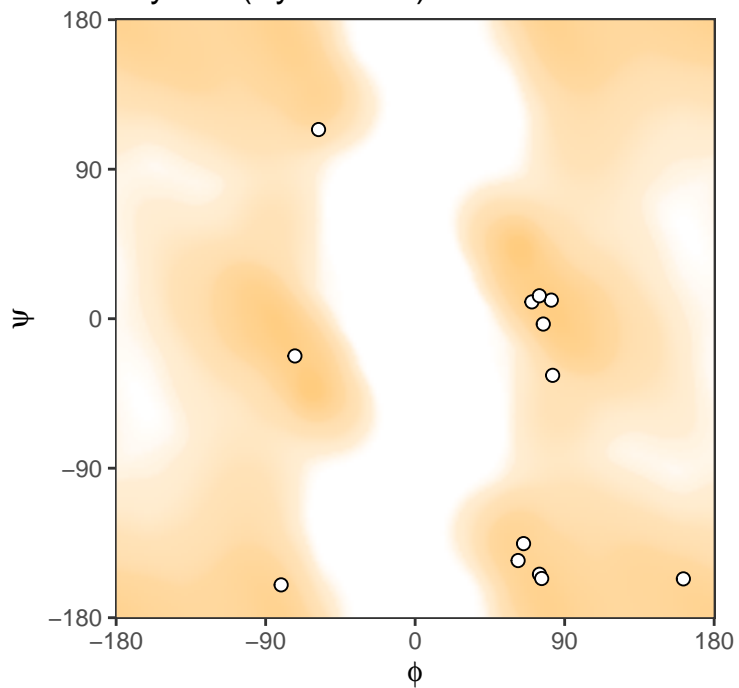

Pre-Proline

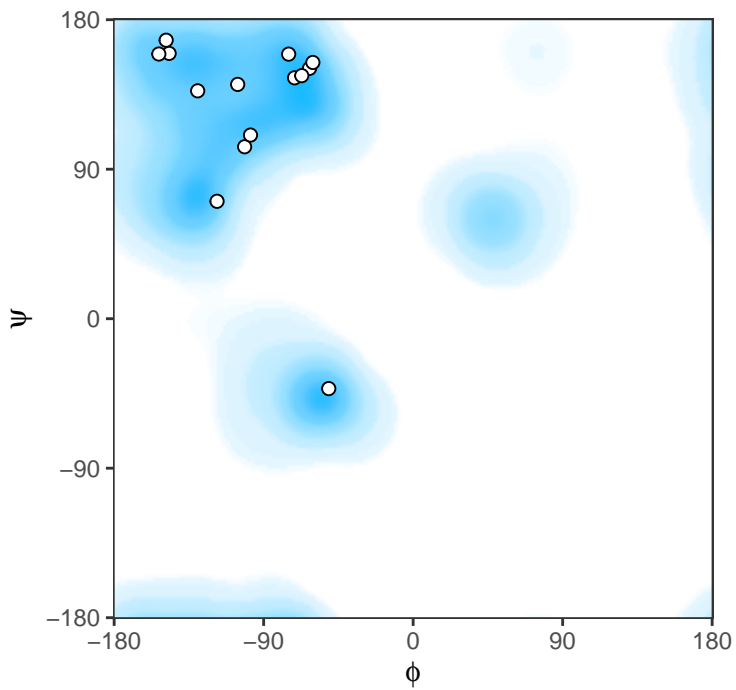

Proline

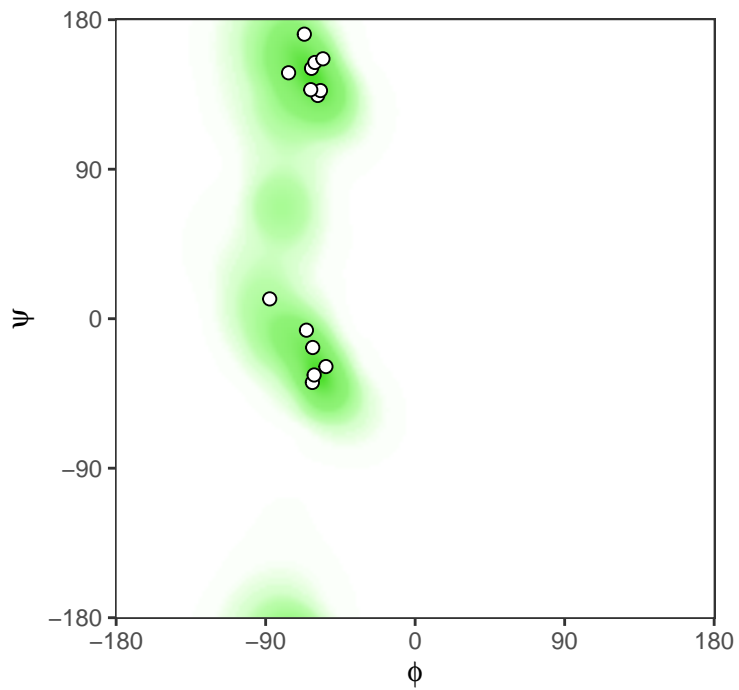

Supplement: Supplementary file 1 [file antioxidants-11-00488-s001.zip › Supplement S7/Soltu.DM.06G012170.6_ramachandran.pdf]

General

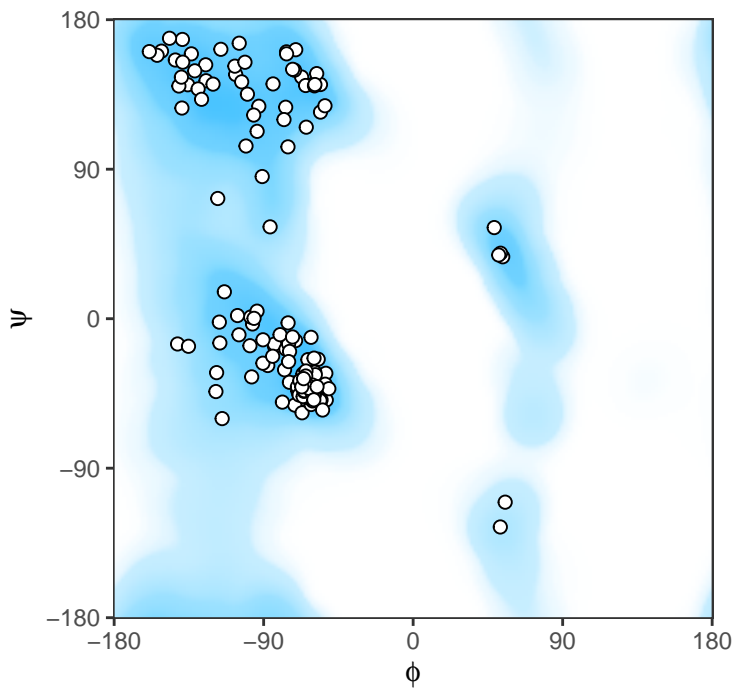

Glycine (Symmetric)

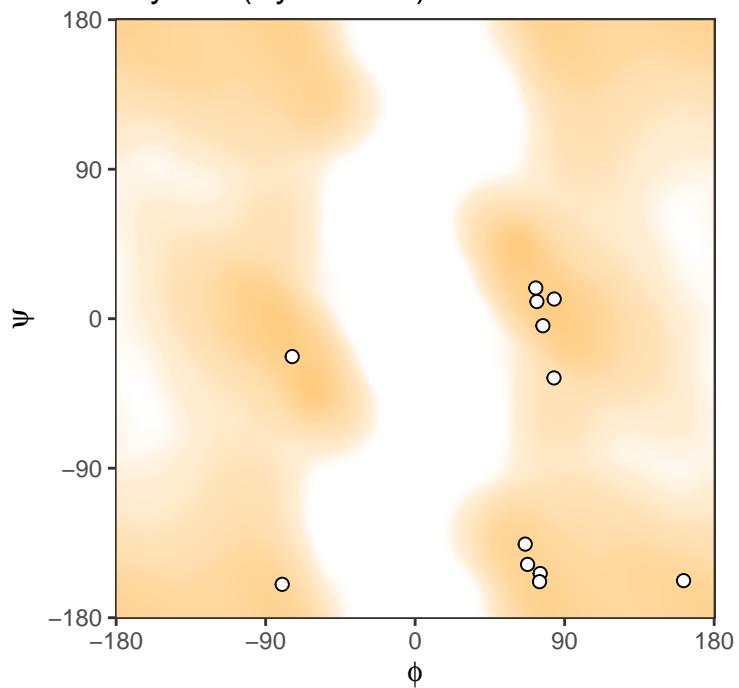

Pre-Proline

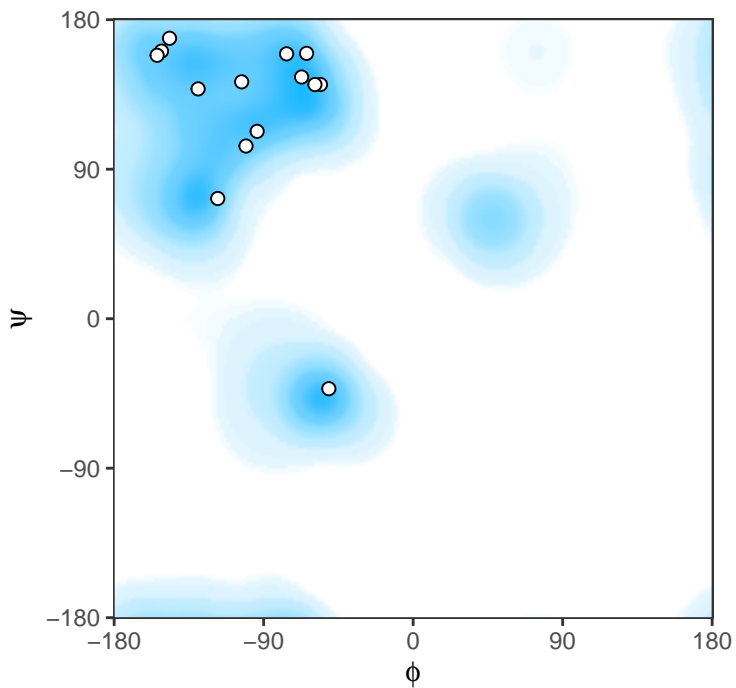

Proline

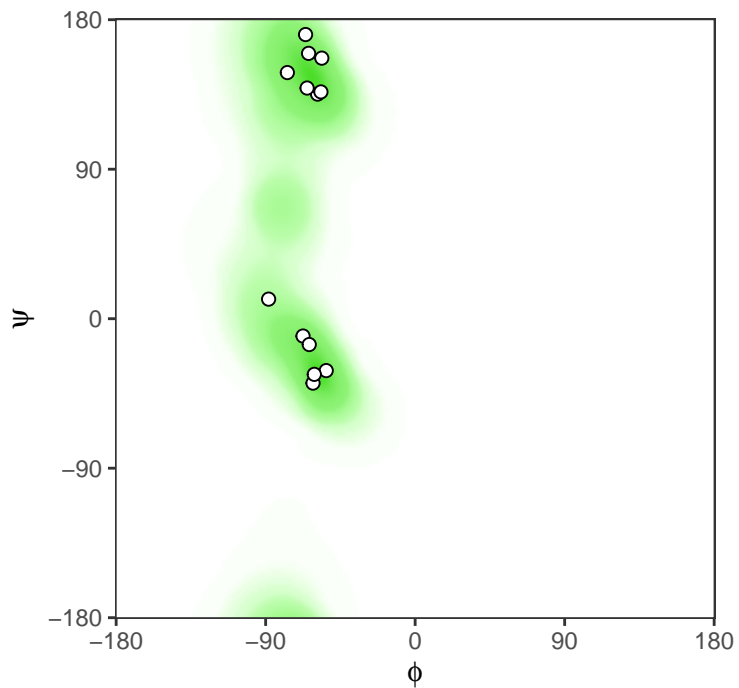

Supplement: Supplementary file 1 [file antioxidants-11-00488-s001.zip › Supplement S7/Soltu.DM.06G012180.3_ramachandran.pdf]

General

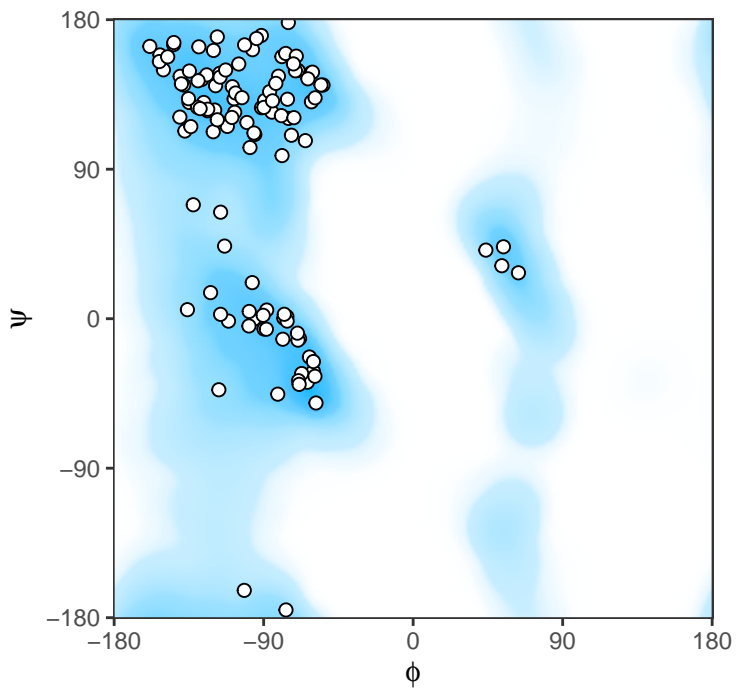

Glycine (Symmetric)

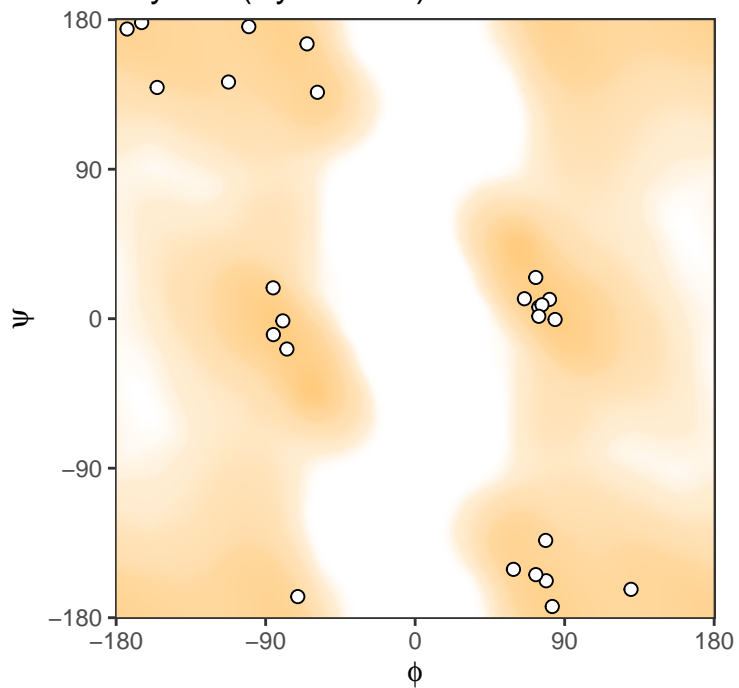

Pre-Proline

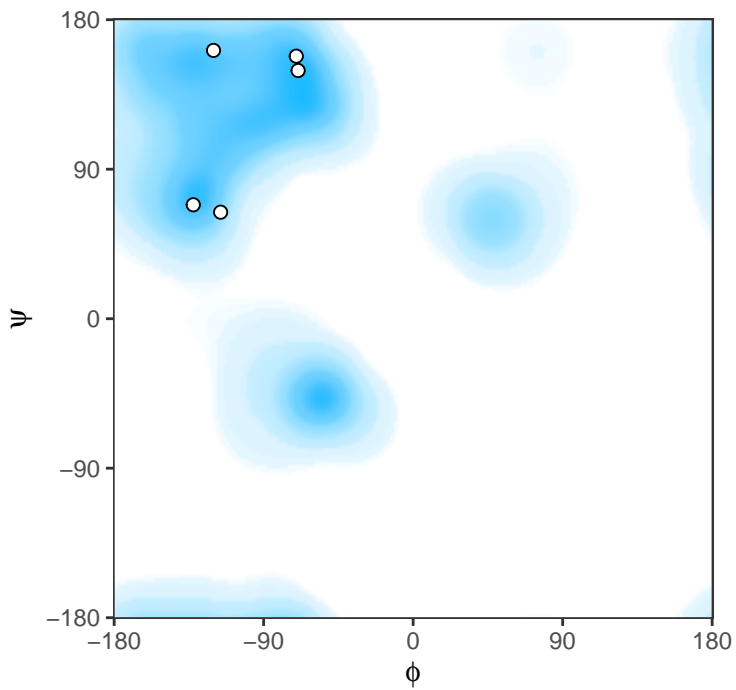

Proline

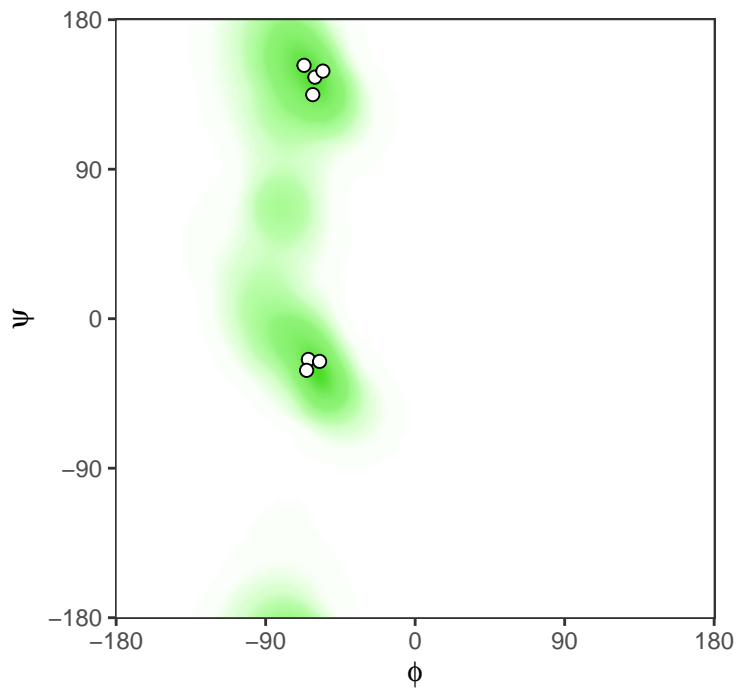

Supplement: Supplementary file 1 [file antioxidants-11-00488-s001.zip › Supplement S7/Soltu.DM.11G020830.1_ramachandran.pdf]

## B

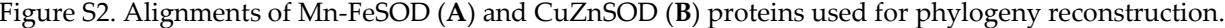

Supplement: Supplementary file 1 [file antioxidants-11-00488-s001.zip › Supplementary S2.pdf]
